# Late-stage MC38 tumours recapitulate features of human colorectal cancer – implications for appropriate timepoint selection in preclinical studies

**DOI:** 10.3389/fimmu.2023.1152035

**Published:** 2023-04-21

**Authors:** Nicholas J. Shields, Estelle M. Peyroux, Angela L. Ferguson, Megan Steain, Silke Neumann, Sarah L. Young

**Affiliations:** ^1^ School of Medical Sciences, Faculty of Medicine and Health, The University of Sydney, Sydney, NSW, Australia; ^2^ Department of Pathology, Otago Medical School, University of Otago, Dunedin, New Zealand; ^3^ Liver Injury and Cancer Program, Centenary Institute, Sydney, NSW, Australia; ^4^ Charles Perkins Centre, University of Sydney, Sydney, NSW, Australia; ^5^ Faculty of Science, University of Canterbury, Christchurch, New Zealand

**Keywords:** colorectal cancer, immunotherapy, syngeneic preclinical models, MC38, immune exclusion, t cell exhaustion, tumour microenvironment

## Abstract

Anti-tumour T cell responses play a crucial role in controlling the progression of colorectal cancer (CRC), making this disease a promising candidate for immunotherapy. However, responses to immune-targeted therapies are currently limited to subpopulations of patients and specific types of cancer. Clinical studies have therefore focussed on identifying biomarkers that predict immunotherapy responses and elucidating the immunological landscapes of different cancers. Meanwhile, our understanding of how preclinical tumour models resemble human disease has fallen behind, despite their crucial role in immune-targeted drug development. A deeper understanding of these models is therefore needed to improve the development of immunotherapies and the translation of findings made in these systems. MC38 colon adenocarcinoma is a widely used preclinical model, yet how it recapitulates human colorectal cancer remains poorly defined. This study investigated the tumour-T cell immune landscape of MC38 tumours using histology, immunohistochemistry, and flow cytometry. We demonstrate that early-stage tumours exhibit a nascent TME, lacking important immune-resistance mechanisms of clinical interest, while late-stage tumours exhibit a mature TME resembling human tumours, with desmoplasia, T cell exhaustion, and T cell exclusion. Consequently, these findings clarify appropriate timepoint selection in the MC38 model when investigating both immunotherapies and mechanisms that contribute to immunotherapy resistance. Overall, this study provides a valuable resource that will enable appropriate application of the MC38 model and expedite the development and clinical translation of new immunotherapies.

## 1 Introduction

Murine tumour models have been crucial for the preclinical development and optimisation of many cancer therapies (1, 2). However, in the case of immunotherapies, progress has been hindered in part by the lack of suitable models that accurately predict successful clinical responses in humans (1). Mounting evidence illustrates that human tumours and the composition of their immune infiltrates are highly heterogeneous (3, 4) and that effective anti-tumour immunity can be hindered by numerous immune evasion and suppression mechanisms (5). Consequently, various clinical studies have focussed on identifying biomarkers and immune signatures that can predict responses to immunotherapy (4, 6–9). In addition, there is an increasing emphasis on elucidating the immunological landscapes of different cancers and cancer subtypes (10–12), even down to the inherent immunological status of individual cancer patients (13). In the context of colorectal cancer (CRC), clinical studies examining the immune landscape of distinct CRC subtypes have occurred at such pace that they have outstripped our understanding of preclinical tumour models and how these relate to human disease. Consequently, a deeper understanding of preclinical tumour models and how these models resemble human CRC is vital for the clinical translation of results obtained in these systems.

The MC38 cell line was originally derived in 1975 from a colon adenocarcinoma induced by subcutaneous injection of dimethylhydrazine in a female C57BL/6 mouse (14). Due to the intact immune system of syngeneic C57BL/6 hosts, this cell line (among others derived from different tumour types) has been extensively used as a transplantable tumour model for investigating anti-tumour immunity and novel immunotherapies (15–17). As in other syngeneic tumour models, MC38 tumour cells are implanted subcutaneously owing to the advantages of this approach – namely the simplicity and reproducibility of inoculation and the ease of monitoring subsequent tumour growth (1, 18). While this approach obviously fails to recapitulate the complexity of colorectal carcinogenesis *in situ*, the MC38 model has been reported as a reliable representation of advanced disease that has escaped immune selection (19). One obvious limitation of this approach is that the immune microenvironment in subcutaneous tissue is significantly different from the gastrointestinal tract (20). Consequently, orthotopic models have been developed using the MC38 cell line, which involve tumour cell implantation in the mouse colon wall (20, 21). This approach is advantageous over subcutaneous inoculation in that it mimics the growth of primary tumours within the colonic microenvironment (1). However, the complexity of surgical implantation procedures limits the feasibility of this approach, while trauma and inflammation arising from the surgical procedure can create a non-physiologic tumour microenvironment (TME) (20, 21). In addition, it is difficult to monitor tumour growth in the mouse colon. Hence, despite its caveats, subcutaneous implantation of the MC38 cell line remains a useful and practical preclinical model for the study of anti-tumour immune responses.

In contrast to the extensive molecular characterisation of human CRC (22–24), contemporary studies have only recently begun to define the features of preclinical tumour models and how they recapitulate human disease (19, 25, 26). Of note, the genomic and transcriptomic landscape of the MC38 cell line has been characterised using whole-exome sequencing, single-nucleotide polymorphism (SNP) array analysis, and RNA sequencing (19). This study found that the MC38 cell line exhibited DNA mismatch-repair (MMR) deficiency and a propensity for C > A/G > T transversions – mutational signatures that are associated with hypermutated human CRC tumours containing defects in the DNA polymerases Pol δ (*POLD1*) and Pol ϵ (*POLE*) (19, 27). Consistent with this notion, the MC38 genome was shown to harbour mutations in *POLD1* and the MMR gene, *MSH3* (19). In terms of known CRC driver mutations present in the MC38 genome, striking similarities to human CRC (23, 24) are present, with genetic alterations in components of the WNT/β-catenin, TGF-β, EGFR and downstream MAPK signalling pathways (19). Consequently, the MC38 cell line has been purported as a valid model for hypermutated and/or microsatellite instability-high (MSI-H) human CRC (19).

In accordance with its characterisation as a valid model of hypermutated/MSI-H human CRC – and consistent with human consensus molecular subtype 1 (CMS1) tumours (28, 29) – the MC38 model is responsive to immune checkpoint inhibition of the PD-1/PD-L1 axis (15, 30). Consequently, MC38 tumours are widely considered as immunogenic and this model has been used extensively for the preclinical development and optimisation of new immunotherapeutic strategies (17, 30, 31). Studies investigating therapeutically-induced immune responses in the MC38 model have shown that these tumours evade anti-tumour immunity through a variety of different mechanisms, including the recruitment of regulatory CD4^+^ T cells (T_REG_) (19), the expression of inhibitory ligands such as PD-L1 by tumour and myeloid cells (15), and by inducing the dysfunction of infiltrating CD8^+^ T cells (32, 33). Importantly, these immune features closely emulate those observed in human CRC tumours and are associated with unfavourable patient outcomes in CRC and other malignancies (34–38). However, despite widespread use of the MC38 model in cancer immunotherapy research, spontaneous or ‘naturally-occurring’ T cell responses against this cell line (that occur in the absence of therapeutic interventions) are poorly characterised. Furthermore, the spatiotemporal dynamics of T cell infiltration in MC38 tumours during their establishment and progression is largely undefined. Given the importance of the immune landscape in human CRC tumours – both for patient prognosis and predicting therapeutic responses to immunotherapy (9, 39, 40) – a deeper understanding of the immune microenvironment in MC38 tumours is needed.

This study therefore investigated the tumour-T cell immune landscape in the MC38 model, using histology, immunohistochemistry (IHC) and flow cytometry to longitudinally profile endogenous T cell responses in outgrowing tumours. We demonstrate that the progression of MC38 tumours following subcutaneous implantation is a dynamic process, with profound changes in the nature of T cell infiltration and the structural architecture of the MC38 TME. Our findings demonstrate the importance of appropriate timepoint selection when investigating novel immunotherapies and mechanisms of immunotherapy resistance in this model. Collectively, this study provides a resource that may improve the translation of effective immunotherapies to human patients.

## 2 Materials and methods

### 2.1 Mice and cell culture

6- to 10-week-old female C57BL/6 mice were obtained from the Hercus-Taieri Research Unit (HTRU) at the University of Otago, Dunedin, New Zealand. All mice were maintained under specific pathogen-free conditions and all experiments were conducted in accordance with protocols approved by the University of Otago Animal Ethics Committee. The MC38 colon adenocarcinoma cell line was maintained in DMEM media (Gibco, Waltham, MA, USA) supplemented with 10% heat-inactivated FCS (Moregate Biotech, Hamilton, NZ), 100 U/mL penicillin and 100 mg/mL streptomycin (Gibco) and harvested using DPBS (Gibco) containing 0.02% EDTA. Cells were routinely tested for mycoplasma contamination using a commercial mycoplasma detection kit (QuickTest, Bimake, Houston, TX, USA) in accordance with the manufacturer’s instructions. Primary murine cells were cultured in RPMI 1640 medium (Gibco) supplemented with 10% FCS (Moregate Biotech), 100 U/mL penicillin, 100 U/mL streptomycin and 50 μM 2-mercaptoethanol (2-ME, all from Gibco). Bone marrow-derived dendritic cells (BMDCs) were generated by culturing bone marrow progenitors (isolated from C57BL/6 mice) with 20 ng/mL recombinant murine GM-CSF (BioLegend, San Diego, CA, USA) as previously described (41). To assess PD-L1 and MHC class I (MHC-I, H-2K^b^) expression *in vitro*, MC38 tumour cells were cultured in the presence or absence of 10 ng/mL recombinant murine interferon-γ (IFN-γ, Prospec-Tany TechnoGene, Rehovot, Israel). At indicated timepoints, cells were stained with PerCP-Cy5.5 anti-H-2K^b^ and PE-Cy7 anti-PD-L1 antibodies (both from BioLegend, refer to **Supplementary Table 1**) and assessed by flow cytometry.

### 2.2 MC38 tumour model

MC38 tumour cells were harvested, washed extensively, and resuspended in DPBS (Gibco). C57BL/6 mice were inoculated with 1 × 10^5^ MC38 cells (suspended in 100 µL DPBS) by subcutaneous (s.c.) injection in the right hind flank. In some experiments, mice were inoculated with 0.5 × 10^6^ or 1.0 × 10^6^ MC38 cells. Injections were performed using 0.5 mL insulin syringes with 0.33 mm (29G) x 12.7 mm needles (BD, Franklin Lakes, NJ, USA). Tumour size (mm^2^) was monitored daily or every two days using a digital calliper and calculated as the product of the largest perpendicular diameters. Mice were euthanised once tumours reached an area of 150 mm^2^.

### 2.3 Histology, immunohistochemistry and image acquisition

Tumours were fixed in 10% neutral buffered formalin (Sigma-Aldrich) for 24 hours at room temperature, dehydrated, and embedded in paraffin. Sequential 3 μm tissue sections were cut from tumours at the region of greatest diameter and mounted on slides for staining procedures.

Haematoxylin and eosin (H&E) and reticular fibre staining was performed according to standard procedures by the Otago Micro and Nanoscale Imaging (OMNI) Histology Unit (University of Otago). For H&E staining, Gill II haematoxylin and eosin were obtained from Leica Biosystems, Wetzlar, Germany. Potassium permanganate, oxalic acid, ferric ammonium sulphate, ammoniacal silver solution, gold chloride, sodium thiosulphate and Nuclear Fast Red solution used for reticular fibre staining were all obtained from Sigma-Aldrich. Stained sections were dehydrated through ethanol and xylene, and then coverslipped with DPX mountant (Sigma-Aldrich).

Immunohistochemistry (IHC) for CD3, CD4, CD8 and CD31 was performed by the OMNI Histology Unit (University of Otago) on a BOND RX^m^ automated stainer (Leica Microsystems, Bannockburn, IL, USA). Staining was performed using the BOND Polymer Refine Detection kit (Leica Biosystems) according to optimised protocols. Briefly, 3 µm tumour sections were deparaffinised and rehydrated, before blocking endogenous peroxidase activity with 3 – 4% (v/v) hydrogen peroxide (Peroxidase Block, Leica Biosystems). Heat-induced epitope retrieval was performed using either BOND Epitope Retrieval Solution 1 (ER1, citrate-based, pH 6.0) or BOND Epitope Retrieval Solution 2 (ER2, EDTA-based, pH 9.0; both from Leica Biosystems). After blocking in 2 – 10% (w/v) BSA, sections were incubated with primary antibodies for optimised durations. Primary antibodies were diluted 1:1000 in BOND Primary Antibody Diluent (Leica Biosystems) or Da Vinci Green Diluent (Biocare Medical, Pacheco, CA, USA) and are listed as follows: recombinant rabbit monoclonal anti-CD31 (clone EPR17259, ab182981, Abcam), rabbit polyclonal anti-CD3 antibody (ab5690, Abcam), recombinant rabbit monoclonal anti-CD4 (clone EPR19514, ab183685, Abcam), and rabbit monoclonal anti-CD8 (clone SP16, ab101500, Abcam). Following the manufacturer’s instructions (BOND Polymer Refine Detection kit, Leica Biosystems), primary antibody binding was visualised using 3,3’-diaminobenzidine (DAB) tetrahydrochloride hydrate and counterstained with haematoxylin. The sections were then washed, dehydrated, and coverslipped using DPX mountant (Sigma-Aldrich). In all IHC staining, murine spleen tissue was used for positive anatomical controls, and tumour tissue with primary antibodies omitted was used for negative controls (no-primary controls).

Whole slide images of stained sections were generated using an Aperio ScanScope automated scanner (Leica Biosystems) using the 20× (0.50 µm/pixel resolution) and ×40 (0.25 µm/pixel resolution) objectives to produce uniplanar digital slides. Aperio ImageScope software (Leica Biosystems) was used to extract images of entire tumour sections for subsequent analysis (TIFF format, full resolution at 20× magnification). Quantitative evaluation of IHC images was performed according to published methodology (42) using ImageJ software (NIH) and is described in **Supplementary Material** (**Supplementary Figures 1–3**).

### 2.4 Enzyme-linked immunosorbent assay for total TGF-β1

To generate tumour-conditioned media, MC38 cell culture supernatants were harvested after 24 and 48 hours and centrifuged to remove tumour cells. Tumour homogenate samples were prepared from MC38 tumours excised 14- and 21-days post-inoculation. Briefly, resected tumours were weighed and then homogenised in controlled volumes of RPMI media, before collecting the supernatants after centrifugation. Total TGF-β1 levels in MC38 tumour-conditioned media and resected tumour homogenates were quantified using a murine TGF-β1 ELISA kit (Invitrogen, Carlsbad, CA, USA) according to the manufacturer’s instructions. RPMI media (no FCS) and complete DMEM media (containing 10% FCS) were used as background controls for tumour homogenates and tumour-conditioned media samples, respectively. Absorbance was measured at 450 and 570 nm using a Victor X4 plate reader (Perkin Elmer) and absorbance values for samples were blank and background subtracted. TGF-β1 concentrations (pg/mL) were interpolated from a standard curve generated using Prism 8.0.1 software (GraphPad). For tumour homogenate samples, the total amount of TGF-β1 per tumour (pg) was normalised to tumour mass (pg/mg).

### 2.5 Tissue processing and tumour-infiltrating lymphocyte isolation

Mice were euthanised after 7, 14 and 21 days (post tumour inoculation) and single-cell suspensions prepared from tumours and tumour-draining inguinal lymph nodes (LNs). Mechanical dissociation was employed for the isolation of TILs, following a validated protocol with slight alterations (43). Briefly, resected tumours were weighed and then processed into <1 mm^3^ pieces using a scalpel in cold RPMI 1640 media. Tumour fragments were transferred into mechanical dissociator C tubes (Miltenyi Biotec) and homogenised on a GentleMACS dissociator (Miltenyi Biotec). Cell strainers and C tubes were rinsed to collect residual cells and tumour homogenates were passed through 70 µm cell strainers, which were rinsed repeatedly with cold media. Filtrates were collected and centrifuged, before the resulting cell pellets were resuspended in DPBS and layered onto Ficoll-Paque PLUS (GE Healthcare, Uppsala, Sweden) for density gradient centrifugation (as per the manufacturer’s instructions). Cells were collected from the high-density solution interface using a transfer pipette and washed twice in DPBS containing 5% FCS at 4°C. LNs were processed on ice in cold DPBS containing 5% FCS and homogenised by forcing the tissue through 70 µm nylon cell strainers. Resulting homogenates were then passed through 40 µm nylon cell strainers to remove residual tissue. Isolated tumour and LN cells were resuspended in media or DPBS at the appropriate density for subsequent procedures.

### 2.6 Flow cytometry and antibodies

Single-cell suspensions were stained using the Zombie Yellow Fixable Viability kit (BioLegend) or the LIVE/DEAD Fixable Violet Dead Cell Stain kit (Invitrogen) to exclude dead cells. Non-specific antibody binding was blocked using anti-mouse CD16/CD32 Fc Block solution (BD Biosciences, San Diego, CA, USA) following the manufacturer’s protocol. For cell surface staining, cells were incubated with fluorophore-conjugated antibodies in FACS buffer (PBS with 0.1% w/v BSA, 0.01% w/v sodium azide, 0.2 mM Na_2_ EDTA, pH 7.4) for 15 minutes at 4°C. Intracellular staining for transcription factors and cytokines was performed using the True-Nuclear Buffer Set (BioLegend) according to the manufacturer’s protocol. All antibodies used for flow cytometry are detailed in **Supplementary Table 1**. After antibody staining, cells were washed extensively in FACS buffer and analysed on a Gallios flow cytometer (10 colour configuration equipped with blue (488 nm), red (633 nm) and violet (405 nm) lasers) using Kaluza Acquisition and Analysis software (all from Beckman Coulter, Fullerton, CA, USA). A standard gating strategy was employed for data analysis, involving doublet exclusion based on forward scatter (FSC) and side scatter (SSC) characteristics, and the exclusion of dead cells based on viability staining. Populations of interest were subjected to further analysis after gating on fluorescence parameters for lineage-specific markers (e.g. CD3 and CD4/CD8). Fluorescence-minus-one (FMO) controls were used to establish gating thresholds for positivity (44).

### 2.7 *Ex vivo* T cell stimulation assays

For phorbol 12-myristate 13-acetate (PMA)/ionomycin stimulation, CD3^+^ T cells were enriched from D14 and D21 MC38 tumour dissociates using the Pan T Cell Isolation kit (negative selection), autoMACS columns, and an autoMACS Pro automated separator according to the manufacturer’s instructions (all from Miltenyi Biotec). Following enrichment, cells were rested overnight in complete medium supplemented with 5 ng/mL recombinant murine interleukin-7 (IL-7, Prospec-Tany TechnoGene) and then stimulated with 50 ng/mL PMA and 500 ng/mL ionomycin (both from Sigma-Aldrich) or left untreated (unstimulated controls). Cells were incubated for 5 hours in the presence of 5 μg/mL brefeldin A (BFA; BioLegend) and then harvested, washed, and stained for flow cytometric analysis. Intracellular staining for IFN-γ, IL-2, IL-10, TNF and granzyme B (GzmB) was performed using the True-Nuclear Buffer Set (BioLegend) according to the manufacturer’s protocol.

For peptide stimulation, CD8^+^ T cells were isolated from D14 and D21 MC38 tumours and tumour-draining lymph nodes (TdLNs) using the CD8α+ T cell isolation kit (Miltenyi Biotec) and then rested overnight in complete medium supplemented with 5 ng/mL rmIL-7 (Prospec-Tany TechnoGene). BMDCs were either left untreated (no peptide) or pulsed with 1 µg/mL of the following peptides (all from Mimotopes, Mulgrave, VIC, AU): MuLV p15E_604-611_ (KSPWFTTL), Adpgk_R304M_ (ASMTNMELM), Dpagt1_V213L_ (SIIVFNLL), or OVA_257–264_ (SIINFEKL; irrelevant peptide). BMDCs were incubated for 3 hours, washed, and then plated in triplicate wells per condition (10^5^ cells/well). CD8^+^ T cells were added to BMDCs at a 10:1 ratio and incubated for 6 hours in the presence of 5 μg/mL BFA (BioLegend). Cells were then harvested, washed, and stained for flow cytometric analysis. Antigen-specific CD8^+^ T cell responses were detected *via* intracellular staining for TNF and IFN-γ as described above.

### 2.8 Statistical analysis

All statistical analyses were performed using GraphPad Prism 8.0.1 software (GraphPad Software, La Jolla, CA, USA). Specific statistical tests performed are indicated in corresponding figure legends. *p*-values of <0.05 were considered statistically significant and significance levels are annotated as: *p ≤ 0.05; **p ≤ 0.01; ***p ≤ 0.001; ****p ≤ 0.0001. All graphs depict the mean ± the standard error of the mean (SEM; error bars).

## 3 Results

### 3.1 Growth kinetics and histological characteristics of MC38 colon adenocarcinoma *in vivo*

**Figure 1:**
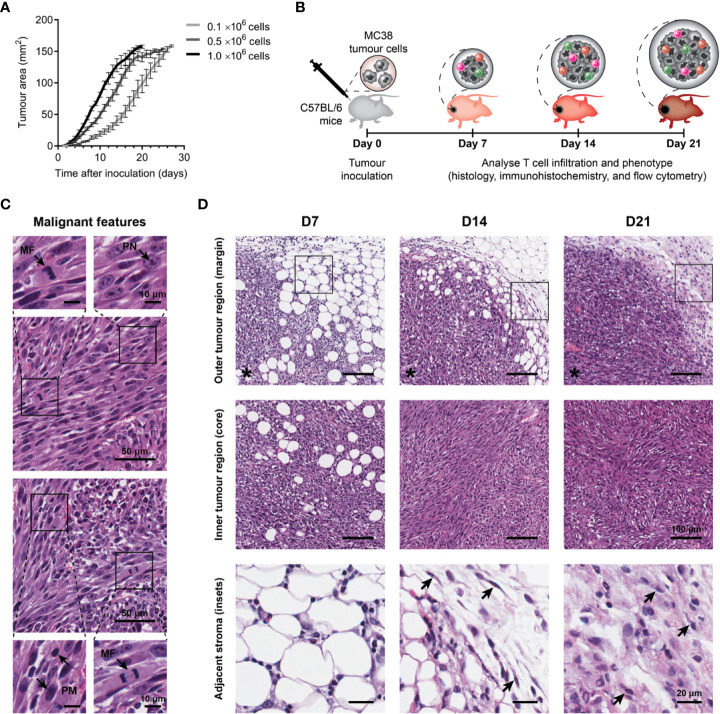
Growth kinetics and histological features of MC38 tumours. **(A)** Growth kinetics of MC38 tumours *in vivo*. Syngeneic C57BL/6 mice were inoculated with indicated numbers of MC38 cells by subcutaneous injection at the right hind flank (n = 10 per group). Growth curves depict the mean and standard error of the mean (SEM, error bars). **(B)** Study design to characterise T cell infiltration. MC38 tumours were resected 7-, 14- and 21-days post-inoculation and processed for histology, immunohistochemistry, or flow cytometry. **(C)** Histological features in haematoxylin and eosin (H&E) stained MC38 tumour sections. Arrows indicate characteristics of high-grade neoplasms, including hyperchromatic nuclei containing multiple, prominent nucleoli (PN; arrow, top left inset), mitotic figures (MF; arrows, bottom left and top right insets) and pleomorphism (PM; arrows, bottom right inset). **(D)** H&E images depicting structural changes in the outer (top row) and inner (middle row) regions of MC38 tumours during outgrowth (D7, D14 and D21) and high magnification images of adjacent stroma at the tumour margin (bottom row, ROI shown in top row). Arrows indicate stromal cells with morphological features typical of normal fibroblasts in D14 tumours (thin, wavy, spindle-shaped cells), and cancer-associated fibroblasts (CAFs) in D21 tumours (large cells with plump nuclei). Asterisks (*) denote the side of the tumour in images of the tumour margin. Scale bar dimensions are shown at the right of each row (10, 20, 50 and 100 μm).

Firstly, the optimal number of tumour cells required to reproducibly induce slow growing subcutaneous tumours was determined. In our hands, inoculation with 0.1×10^6^ MC38 cells produced consistent engraftment and desirable tumour growth kinetics (compared to higher inoculums), with tumour size reaching ≥150 mm^2^ after a mean of 23 days (**Figure 1A**).

To understand the evolution of the MC38 TME over progressive outgrowth, tumours resected at days 7, 14 and 21 (after inoculation; D7, D14 & D21) were processed for analyses (**Figure 1B**). Across all timepoints, H&E staining revealed MC38 tumours exhibited hallmarks of high-grade neoplasms *in vivo*, with frequent mitotic figures and pleomorphic, hyperchromatic nuclei containing prominent nucleoli (**Figure 1C**). Changes in stromal architecture during tumour progression were apparent, particularly at the invasive tumour margin (**Figure 1D**). At the earliest timepoint (D7), MC38 tumours displayed diffuse margins with tumour cells infiltrating surrounding adipose tissue (outer tumour region; **Figure 1D**). D14 MC38 tumour margins were more defined with an increase in extracellular matrix (ECM) deposition, while D21 MC38 tumours displayed well-defined margins exhibiting desmoplasia with pervasive deposition of fibrous ECM (**Figure 1D**, adjacent stroma). Stromal cells embedded within this ECM exhibited morphological characteristics of cancer-associated fibroblasts (CAF), with spindle-shaped profiles and plump nuclei (**Figure 1D**, arrows).

Within the inner tumour region (core), adipocytes were present throughout D7 tumours, while D14 and D21 tumours exhibited dense, hypercellular cores with spindle-shaped tumour cells forming a sarcomatoid-like pattern of haphazard, interlacing bundles (inner tumour region; **Figure 1D**). In established MC38 tumours (D14), focal areas of both ischemic and haemorrhagic necrosis were evident, suggestive of inadequate or defective tumour vasculature (**Supplementary Figure 4A**).

### 3.2 T cell infiltration in MC38 tumours progressively decreases during outgrowth, culminating in an immune-excluded phenotype

**Figure 2:**
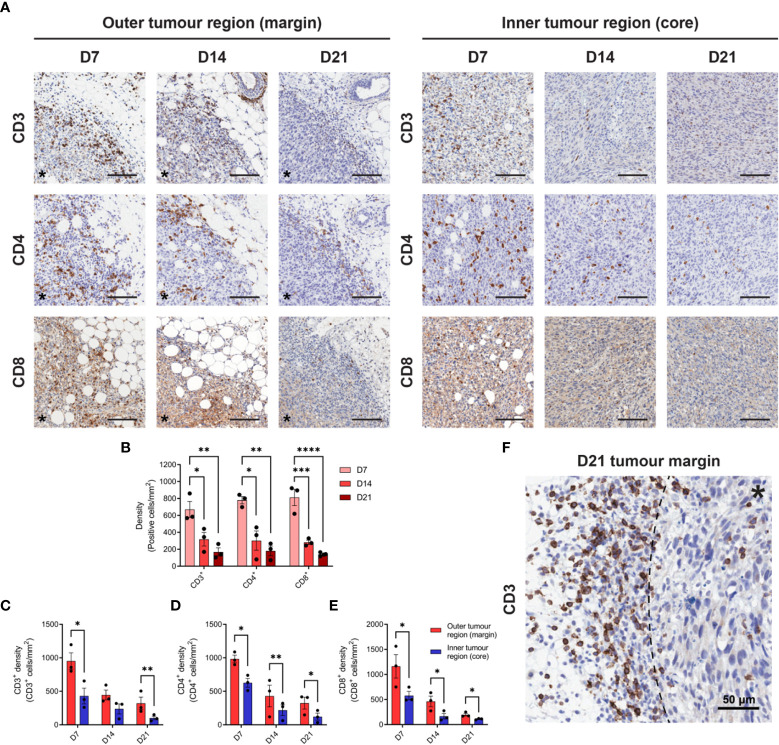
T cell infiltration in MC38 tumours during progressive outgrowth. **(A)** MC38 tumours were resected from mice 7-, 14- and 21-days post-inoculation (n = 3 biological replicates per timepoint) and processed for IHC. Images depict immunohistochemical staining for CD3, CD4, or CD8 (DAB, brown with haematoxylin counterstain) in the outer (left panel) and inner (right panel) regions of MC38 tumours (D7, D14 and D21). All scale bars depict 100 μm. **(B)** Quantification of CD3, CD4, and CD8 infiltration in MC38 tumours (total tumour area) during progressive outgrowth. **(C–E)** Quantification of CD3 **(C)**, CD4 **(D)**, and CD8 **(E)** infiltration in the outer and inner regions of MC38 tumours at indicated timepoints. CD3^+^, CD4^+^, and CD8^+^ density was quantified as the absolute number of positive cells per mm^2^ (positive cells/mm^2^). **(F)** Representative image depicting dense accumulation of CD3^+^ cells at the stromal–tumour interface (dashed line) in late-stage D21 tumours. Asterisks (*) denote the side of the tumour in images of the tumour margin. Graphs represent the mean and standard error of the mean (SEM, error bars). Statistical analyses were performed using one-way ANOVA with *post-hoc* Tukey test to correct for multiple comparisons **(B)** or two-tailed paired Student’s t-tests **(C–E)**. *p ≤ 0.05; **p ≤ 0.01; ***p ≤ 0.001; ****p ≤ 0.0001.

Given the prognostic importance of T cell infiltration in CRC (39), IHC for CD3, CD4 and CD8 was performed to define the spatiotemporal dynamics of T cell infiltration in MC38 tumours. Staining was quantified in two tumour compartments: the ‘outer tumour region’ defined as the area 250 µm inwards from the stromal–tumour interface (tumour margin); and the ‘inner tumour region’ (tumour core) defined as the area encompassed by the outer tumour region. Identical trends for all T cell markers were observed, whereby the density of infiltration by CD3^+^, CD4^+^ and CD8^+^ cells progressively decreased during tumour outgrowth in terms of the total tumour area (**Figures 2A, B**) and within the outer and inner tumour regions (**Figures 2C–E**). Higher densities of CD3^+^, CD4^+^ and CD8^+^ cells were also localised to the outer tumour region compared to the inner tumour core (**Figures 2C–E**). In late-stage D21 tumours, areas of dense T cell accumulation were evident at the tumour margin and adjacent tissue, with pronounced restriction of T cells at the stromal–tumour interface (**Figure 2F**).

### 3.3 MC38 tumours exhibit structurally abnormal vasculature and progressive reticulin fibre deposition at the tumour margin

**Figure 3:**
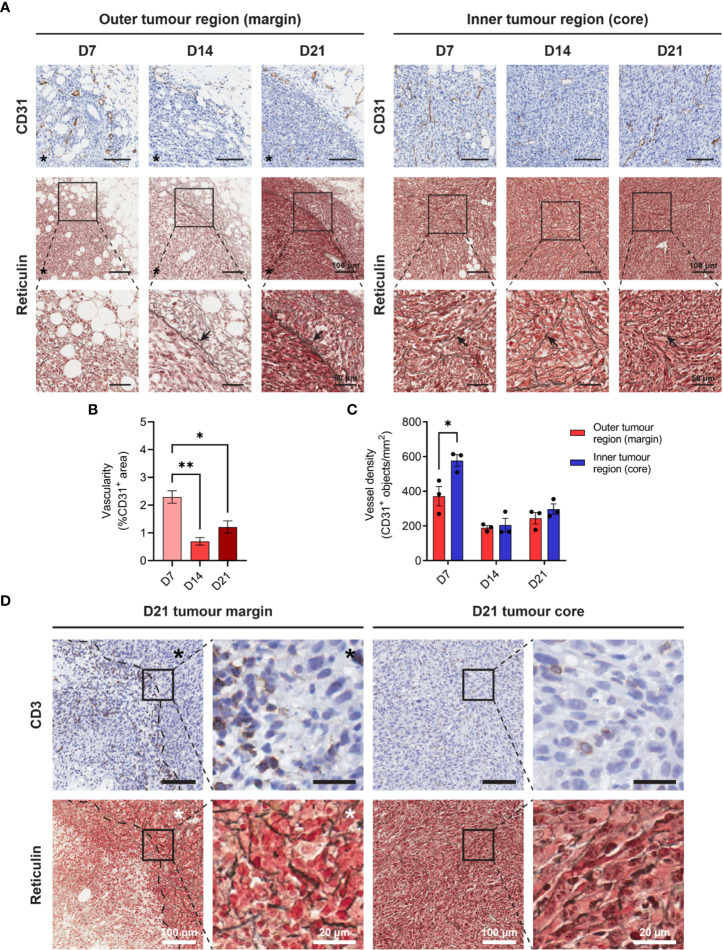
Angiogenesis, reticulin fibrosis, and immune exclusion in MC38 tumours. **(A)** MC38 tumours were resected from mice 7-, 14- and 21-days post-inoculation (n = 3 biological replicates per timepoint) and processed for CD31 IHC and reticulin staining. Images depict immunohistochemical staining for CD31 (brown with haematoxylin counterstain, top row) and reticulin silver staining of argyrophillic ‘reticular fibres’ (black, arrows) with Nuclear Fast Red counterstain (nuclei, red). Scale bars depict 100 μm and 50 μm in low and high magnification images, respectively. **(B)** Vascularity of MC38 tumours determined as the CD31^+^ area proportional to the total tumour area (%CD31^+^ area of total tumour). **(C)** CD31^+^ vessel density in the outer and inner regions of MC38 tumours at indicated timepoints. Vessel density was quantified as the absolute number of CD31^+^ blood vessels per mm^2^ (CD31^+^ objects/mm^2^). **(D)** CD3 IHC and reticulin staining of sequential D21 tumour sections. Top row images depict immunohistochemical staining for CD3 (brown) with haematoxylin counterstain (blue) and bottom row images depict reticulin staining for collagenous ‘reticular fibres’ (black) with Nuclear Fast Red counterstain (red). Asterisks (*) denote the side of the tumour in images of the tumour margin. Scale bars depict 100 μm and 20 μm in low and high magnification images, respectively. Graphs represent the mean and standard error of the mean (SEM, error bars). Statistical analyses were performed using one-way ANOVA with *post-hoc* Tukey test to correct for multiple comparisons **(B)** or two-tailed paired Student’s t-tests **(C)**. *p ≤ 0.05; **p ≤ 0.01.

Increasing metabolic demands of outgrowing tumours requires the generation of new blood vessels (angiogenesis), while T cells are dependent on tumour vasculature to infiltrate and execute their anti-tumour functions. To assess angiogenesis and the microvasculature in MC38 tumours, IHC for the endothelial cell marker CD31 was performed over the course of tumour outgrowth (**Figure 3A**). In early D7 MC38 tumours, vascularisation was pronounced (in terms of both CD31^+^ area and vessel density) compared to intermediate and late-stage tumours (**Figures 3B, C**). There was a trend towards increased vascularisation in D21 versus D14 tumours, however, this was not statistically significant (**Figure 3B**). In relatively avascular D14 tumours, extensive areas of necrosis were present (**Supplementary Figure 4A**), suggesting that tumour vasculature was inadequate for nutrient and oxygen demands at this stage of outgrowth.

The inner core of D7 tumours exhibited higher vessel density compared to the outer regions of these tumours (CD31^+^ objects/mm^2^), while there were no statistical differences in the distribution of CD31^+^ vessels in D14 and D21 tumour regions (**Figure 3C**). Structurally, MC38 tumour vasculature was highly disorganised, with an irregular pattern of interconnection that became increasingly pronounced in late-stage D21 tumours (**Figure 3A**). CD31^+^ vessels in adjacent connective tissue surrounding MC38 tumours appeared dilated but exhibited relatively normal vessel structure characterised by intact CD31^+^ endothelium supported by fine, perivascular connective tissue (**Supplementary Figure 4B**). By contrast, CD31^+^ vessels in both the periphery and centre of D21 MC38 tumours were structurally defective, with aberrant endothelium which was either reduced in thickness or entirely absent (**Supplementary Figure 4B**).

In addition to abnormal tumour vasculature, excessive production of ECM components leading to tumour fibrosis (desmoplasia) can mediate the physical exclusion of T cells from tumours (45). To assess the ECM architecture in the MC38 TME, tumours were subjected to reticulin staining – a non-specific silver impregnation method that visualises argyrophillic ECM fibres (reticulin fibres). Extensive fibrosis was evident throughout the MC38 TME, with reticulin fibres increasing in both frequency and thickness during progressive outgrowth (**Figure 3A**). Distinct differences in the architecture and orientation of reticulin fibres were also observed between the outer and inner tumour regions. Within the tumour core, early tumours (D7) exhibited a delicate meshwork of fine reticulin fibrils, which transitioned to thick, elongated fibres in intermediate (D14) and late-stage (D21) tumours (arrows, **Figure 3A**). In D14 and D21 tumours, these thick intratumoural fibres were orientated along aggregates of interlacing tumour cell bundles, forming a supportive reticular network (**Figure 3A**, insets). At the tumour margin, D7 tumours exhibited a network of fine reticulin fibrils (similar to that in the inner tumour region/core), while D14 and D21 tumours displayed extensive deposition of thick reticulin fibres in the stroma along the invasive front (**Figure 3A**). In contrast to the core of these tumours, these thick reticulin fibres were largely aligned in parallel to the tumour margin, forming a prominent barrier that mirrored the exclusion of infiltrating T cells in D21 tumours (dashed line, **Figure 3D**).

### 3.4 TGF-β secretion and PD-L1 expression are intrinsic immune-suppressive mechanisms present in MC38 tumour cells

**Figure 4:**
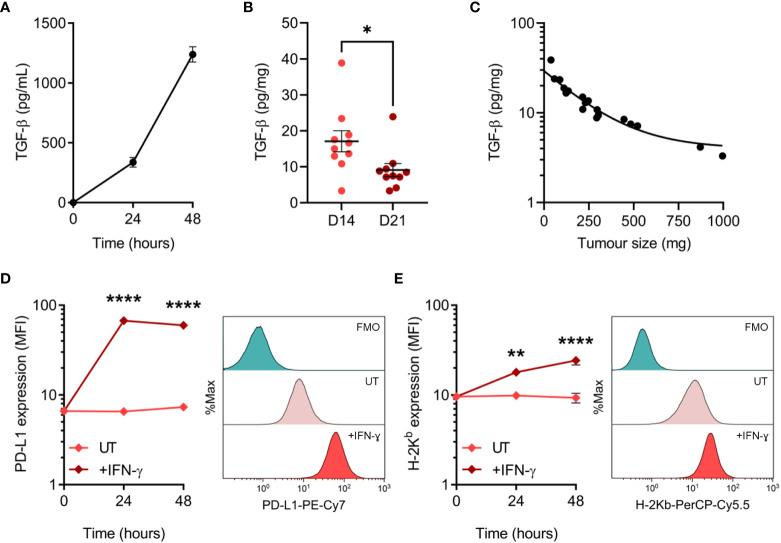
TGF-β secretion and PD-L1 expression by MC38 tumour cells. **(A)** Total TGF-β secreted by MC38 tumour cells over indicated durations *in vitro* (pg/mL). Data are representative of three independent experiments. **(B)** Total TGF-β concentrations in supernatants obtained from D14 and D21 MC38 tumours (n = 10 biological replicates per timepoint). TGF-β levels were normalised to tumour mass (pg/mg). **(C)** Scatter plot showing the relationship between TGF-β concentrations within MC38 tumours (pg/mg) and tumour size/burden (mg). **(D, E)** PD-L1 and MHC class I (H-2K^b^) expression in untreated and IFN-γ-stimulated (10 ng/mL) MC38 tumour cells (median fluorescence intensity, MFI). Representative histogram overlays (right) depict expression in untreated and IFN-γ-stimulated cells after 48 hours and corresponding fluorescence-minus-one (FMO) controls. Data are representative of three independent experiments. Graphs represent the mean and standard error of the mean (SEM, error bars). Statistical analyses were performed using two-tailed unpaired Student’s t-test **(B)**, or two-way ANOVA with *post-hoc* Sidak test to correct for multiple comparisons (comparing IFN-γ-treated samples to untreated controls; **(D, E)**). *p ≤ 0.05; **p ≤ 0.01; ****p ≤ 0.0001.

Tumour cells employ a variety of mechanisms to evade anti-tumour immunity, including the secretion of cytokines and expression of inhibitory cell surface molecules. To elucidate potential mechanisms utilised by MC38, we assessed TGF-β secretion and PD-L1 expression – two well-established immune-suppressive and evasive mechanisms of clinical interest. Secretion of TGF-β by MC38 tumour cells *in vitro* was quantified in tumour-conditioned media, while *ex vivo* TGF-β within the MC38 TME was determined from resected MC38 tumour supernatants. TGF-β was detected in MC38-conditioned media and increased with the duration of culture, indicating secretion of this cytokine by MC38 tumour cells (**Figure 4A**). TGF-β was also abundant in the MC38 TME but decreased in D21 tumours compared to D14 tumours (normalised to tumour mass, pg/mg; **Figure 4B**). Interestingly, there was a relationship between TGF-β levels and the size of MC38 tumours, wherein TGF-β concentrations exponentially decayed with increasing tumour burden (**Figure 4C**).

PD-L1 can be constitutively expressed by tumour cells or induced by exposure to interferons – in particular IFN-γ produced by tumour-specific T cells (46, 47). Hence, while IFN-γ promotes tumour cell recognition *via* the upregulation of MHC-I (48), simultaneous upregulation of PD-L1 can suppress T cell-mediated killing in a process known as adaptive immune resistance (47). Therefore, expression of PD-L1 and MHC-I (H-2K^b^) was assessed at both baseline and in response to IFN-γ exposure. MC38 cells constitutively expressed PD-L1 *in vitro*, which was markedly upregulated in response to IFN-γ and peaked in expression after 24 hours (**Figure 4D**). Similarly, H-2K^b^ expression increased dramatically in response to IFN-γ but exhibited differing kinetics with expression peaking after 48 hours (**Figure 4E**). These results suggest that TGF-β secretion and PD-L1 upregulation are potential mechanisms utilised by MC38 tumour cells to evade anti-tumour immunity.

### 3.5 Dynamic changes in the composition of tumour-infiltrating T cells during MC38 tumour development

**Figure 5:**
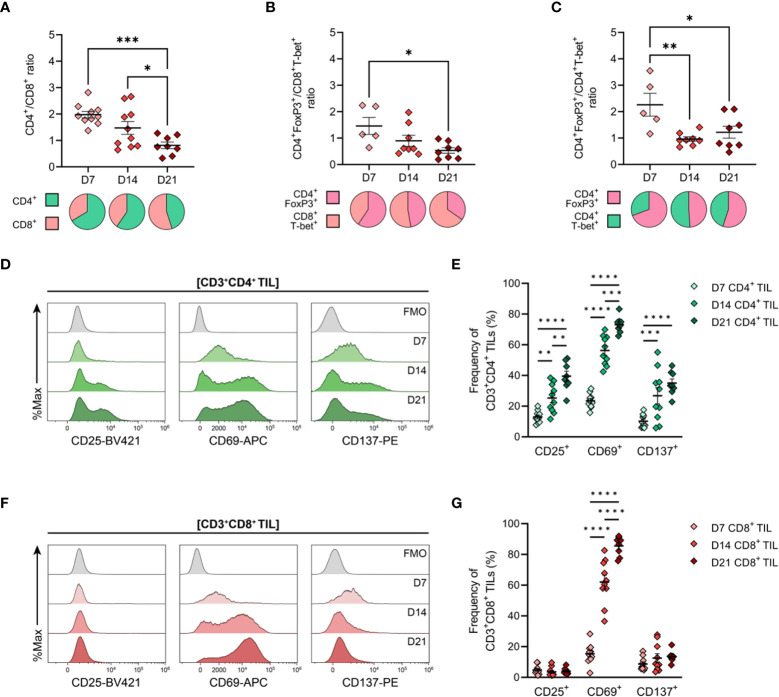
General features of T cell infiltration in MC38 tumours and expression of activation markers by CD4^+^ and CD8^+^ TILs. MC38 tumours were resected 7-, 14- and 21-days post-inoculation (D7, D14 and D21; n = 5-10 biological replicates per timepoint) and mechanically dissociated. Tumour-infiltrating lymphocytes (TILs) were then isolated from tumour homogenates and analysed by flow cytometry. **(A–C)** Graphs depict the ratio of CD3^+^CD4^+^ to CD3^+^CD8^+^ TILs (CD4^+^/CD8^+^ ratio, **(A)**, FoxP3^+^CD3^+^CD4^+^ to T-bet^+^CD3^+^CD8^+^ TILs (CD4^+^ T_REG_/CD8^+^ T_EFF_ ratio, **(B)**) and FoxP3^+^CD3^+^CD4^+^ to T-bet^+^CD3^+^CD4^+^ TILs (CD4^+^ T_REG_/CD4^+^ T_H_1 ratio, **(C)**) during tumour outgrowth (D7, D14 and D21). All ratios were calculated as the quotient of the absolute number of events collected for each of the indicated populations. Pie charts depict relative proportions of indicted populations. **(D, F)** Representative histogram overlays depicting the expression of activation markers (CD25, CD69 and CD137) by total CD3^+^CD4^+^ (**(D)**, green) and CD3^+^CD8^+^ (**(F)**, red) tumour-infiltrating lymphocytes (TIL) isolated from MC38 tumours 7-, 14- and 21-days post-inoculation (D7, D14 and D21). Corresponding fluorescence-minus-one controls (FMO, grey) are also shown. **(E, G)** Frequencies of total CD3^+^CD4^+^ (**(E)**, green) and CD3^+^CD8^+^ (**(G)**, red) TIL expressing indicated activation markers during MC38 tumour outgrowth (D7, D14 and D21). All data are representative of 5-10 biological replicates per timepoint. Graphs represent the mean and standard error of the mean (SEM, error bars). Statistical analyses were performed using one-way **(A–C)** or two-way **(E, G)** ANOVA with *post-hoc* Tukey test to correct for multiple comparisons. *p ≤ 0.05; **p ≤ 0.01; ***p ≤ 0.001; ****p ≤ 0.0001.

Having determined the spatiotemporal dynamics of T cell infiltration in MC38 tumours, the phenotype and functional orientation of these cells were characterised. Changes in the composition and phenotypes of tumour-infiltrating lymphocytes (TILs) were examined by performing flow cytometric analysis on tumour dissociates isolated throughout the course of MC38 tumour progression (D7, D14 & D21; **Figure 1B**). Relative ratios of various T cell subsets in MC38 tumours were examined as a surrogate marker for the inflammatory status of tumour infiltrates. During MC38 tumour outgrowth, tumour infiltration by CD8^+^ T cells markedly increased relative to CD4^+^ T cells (decreasing CD4^+^/CD8^+^ ratio, **Figure 5A**), with the proportion of CD8^+^ effectors (T_EFF_) increasing relative to regulatory CD4^+^ T cells (T_REG_; decreasing CD4^+^FoxP3^+^/CD8^+^T-bet^+^ ratio, **Figure 5B**). Similarly, the proportion of CD4^+^ T helper 1 (T_H_1) effectors increased relative to regulatory CD4^+^ T_REG_ cells in intermediate (D14) and late stage (D21) tumours (decreasing CD4^+^FoxP3^+^/CD4^+^T-bet^+^ ratio, **Figure 5C**). These data suggest that the T cell infiltrate in MC38 tumours becomes increasing inflammatory over the course of tumour progression, with an increase in the proportion of effector cell populations and T cell subsets associated with anti-tumour immunity (49).

### 3.6 Activated effector memory (T_EM_) CD4^+^ and CD8^+^ TILs increase during MC38 tumour progression

Next, the activation status of TILs was analysed as a surrogate marker for anti-tumour T cell responses within the endogenous T cell repertoire. Within the CD4^+^ TIL compartment, there was a progressive increase in proportion of cells expressing the T cell activation markers CD25, CD69, and CD137 (4-1BB), consistent with increased infiltration or expansion of activated CD4^+^ T cells within the MC38 TME (D7→D21; **Figures 5D, E**). Notably, CD25 and CD137 were co-expressed by CD4^+^ TILs (data not shown). Over the course of MC38 tumour progression, there was a significant increase in CD8^+^ TILs expressing CD69 (15→86%; **Figures 5F, G**), with these cells also co-expressing PD-1 (data not shown). The majority of CD8^+^ TILs did not express CD25 or CD137 (<5% and <14% of total CD8^+^ TILs, respectively), with CD25^+^ and CD137^+^ CD8^+^ TIL frequencies remaining stable throughout tumour outgrowth (**Figures 5F, G**). Altogether, these data indicate an endogenous T cell response against MC38 tumours, which is characterised by increased recruitment and/or expansion of activated CD4^+^ and CD8^+^ effectors within the TME.

**Figure 6:**
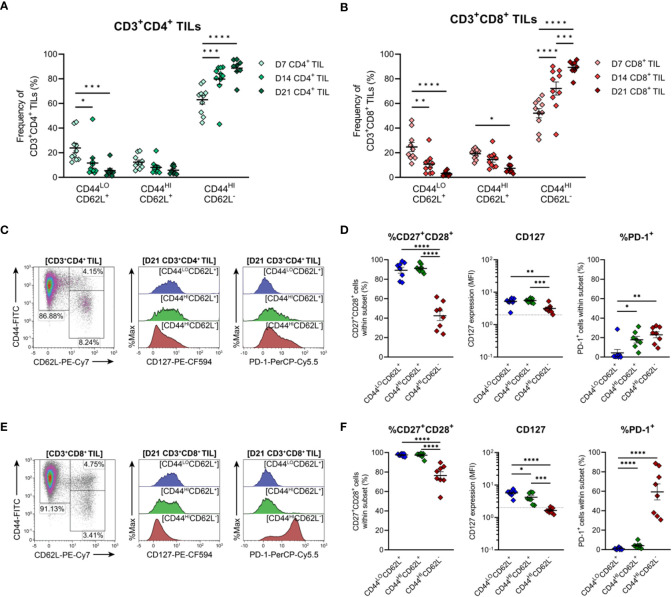
Distribution of CD4^+^ and CD8^+^ T cell subsets infiltrating MC38 tumours. **(A, B)** Frequencies of naïve (CD44^LO^CD62L^+^), central (CD44^HI^CD62L^+^), and effector (CD44^HI^CD62L^−^) memory T cells among CD3^+^CD4^+^ **(A)** and CD3^+^CD8^+^ **(B)** TILs during progressive MC38 tumour outgrowth (D7, D14 and D21). **(C, E)** Representative flow plots depicting the distribution of T cell subsets (based on CD44 and CD62L expression) and CD127 (IL-7Rα) and PD-1 expression in naïve (CD44^LO^CD62L^+^; blue), central memory/T_CM_ (CD44^HI^CD62L^+^; green) and effector memory/T_EM_ (CD44^HI^CD62L^−^; red) T cell subsets within CD3^+^CD4^+^ **(C)** and CD3^+^CD8^+^ **(E)** TIL isolated from D21 MC38 tumours. **(D, F)** Expression of both CD27 and CD28 (%CD27^+^CD28^+^), CD127 (MFI) or PD-1 (%PD-1^+^) within CD44 and CD62L subsets of CD3^+^CD4^+^ **(D)** and CD3^+^CD8^+^ **(F)** TIL isolated from D21 MC38 tumours. In graphs depicting CD127 expression, dotted lines represent the threshold for CD127 positivity determined from fluorescence-minus-one controls. All density plot percentages indicate frequencies as the proportion of the gated population (indicated in bold titles with square brackets). All data are representative of 8-10 biological replicates per timepoint. Graphs represent the mean and standard error of the mean (SEM, error bars). Statistical analyses were performed using two-way **(A, B)** or one-way **(D, F)** ANOVA with *post-hoc* Tukey test to correct for multiple comparisons. *p ≤ 0.05; **p ≤ 0.01; ***p ≤ 0.001; ****p ≤ 0.0001.

To further characterise TILs infiltrating MC38 tumours, the differentiation status of CD4^+^ and CD8^+^ TILs over the course of MC38 tumour progression was investigated. To this end, the distribution of naïve, memory and effector subsets within MC38 TILs was assessed on the basis of CD44 and CD62L expression (50, 51). In both CD4^+^ and CD8^+^ TIL compartments, the proportion of effector memory (T_EM_; CD44^HI^CD62L^−^) cells progressively increased, while the proportion of naïve (T_N_; CD44^LO^CD62L^+^) and central memory (T_CM_; CD44^HI^CD62L^+^) subsets decreased (**Figures 6A, B**). T_EM_ cells were the predominant population throughout the course of tumour outgrowth (>63% and >52% of total CD4^+^ and CD8^+^ TILs, respectively), but early-stage tumours (D7) exhibited substantial infiltration by naïve CD44^LO^CD62L^+^ T_N_ cells (24% and 25% of total CD4^+^ and CD8^+^ TILs, respectively; **Figures 6A, B**). Infiltration by T_CM_ (CD44^HI^CD62L^+^) CD4^+^ and CD8^+^ subsets was consistently low throughout tumour outgrowth (>13% and >20% of total CD4^+^ and CD8^+^ TILs, respectively; **Figures 6A, B**).

To further define these TIL subsets, their expression of CD27, CD28, CD127 (IL-7Rα) and PD-1 was analysed in late-stage D21 MC38 tumours. Within both CD4^+^ and CD8^+^ TIL compartments, naïve (T_N_, CD44^LO^CD62L^+^) and T_CM_ (CD44^HI^CD62L^+^) subsets exhibited high co-expression of costimulatory molecules (CD27 and CD28), and high CD127 expression, consistent with the less-differentiated status of these T cell subsets (**Figures 6C–F**). Indicative of terminal differentiation, CD4^+^ and CD8^+^ T_EM_ (CD44^HI^CD62L^−^) cells displayed low (CD4^+^) or no (CD8^+^) expression of CD127 and down-regulated CD27 expression (**Figures 6D, F**). PD-1 expression was enriched within both CD4^+^ and CD8^+^ T_EM_ TIL subsets (**Figures 6D, F**) but absent in less-differentiated CD8^+^ T_N_ (CD44^LO^CD62L^+^) and T_CM_ (CD44^HI^CD62L^+^) subsets (**Figure 6F**). Consistent with observations in human CRC (52, 53), these data demonstrate that the progression of MC38 tumours is characterised by increased infiltration of CD4^+^ and CD8^+^ T_EM_ cells (as a proportion of total CD4^+^ and CD8^+^ T cells). Furthermore, in late-stage D21 tumours, a subset of these cells within the CD8^+^ TIL compartment exhibit features of terminal differentiation.

### 3.7 Infiltration of MC38 tumours by CD4^+^ T_H_1 effector cells and suppressive regulatory T cells increases during tumour outgrowth

**Figure 7:**
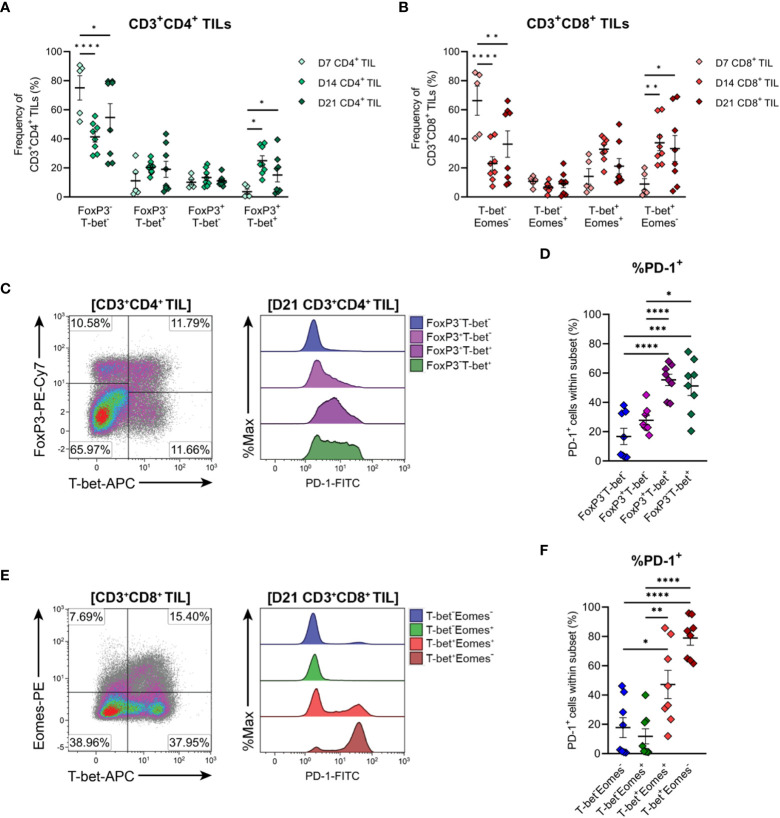
Differentiation status of CD4^+^ and CD8^+^ T cells infiltrating MC38 tumours. **(A)** Frequencies of T-bet and FoxP3 subsets within total CD3^+^CD4^+^ TIL. **(B)** Frequencies of T-bet and Eomes subsets within total CD3^+^CD8^+^ TIL. **(C)** Representative flow plots depicting the distribution of T-bet and FoxP3 subsets and their expression of PD-1 in CD3^+^CD4^+^ TIL isolated from D21 MC38 tumours. **(D)** Expression of PD-1 (%PD-1^+^) within FoxP3 and T-bet subpopulations of D21 CD3^+^CD4^+^ TIL. **(E)** Representative flow plots depicting the distribution of T-bet and Eomes subsets and their expression of PD-1 in CD3^+^CD8^+^ TIL isolated from D21 MC38 tumours. **(F)** Expression of PD-1 (%PD-1^+^) within T-bet and Eomes subpopulations of D21 CD3^+^CD8^+^ TIL. All density plot percentages indicate frequencies as the proportion of the gated population (indicated in bold titles with square brackets). All data are representative of 8-10 biological replicates per timepoint. Graphs represent the mean and standard error of the mean (SEM, error bars). Statistical analyses were performed using two-way **(A, B)** or one-way **(D, F)** ANOVA with *post-hoc* Tukey test to correct for multiple comparisons. *p ≤ 0.05; **p ≤ 0.01; ***p ≤ 0.001; ****p ≤ 0.0001.

The functional polarisation of MC38 TILs was then assessed by examining expression of the T_H_1/cytotoxic T lymphocyte (CTL) transcription factors, T-bet and Eomes, and FoxP3, the master regulator of T_REG_ function. CD4^+^ TILs exhibited heterogeneous expression of both T-bet and FoxP3 in established tumours (D14 & D21), while CD4^+^ TILs in early-stage tumours (D7) were mainly FoxP3^-^T-bet^-^ (75% of total CD4^+^ TILs; **Figure 7A**). These data imply that MC38 tumours are initially infiltrated by naïve CD4^+^ T cells, given that a significant proportion of D7 CD4^+^ TILs also exhibit a naïve CD44^LO^CD62L^+^ phenotype (**Figure 6A**).

As tumours progressed, the frequency of CD4^+^ FoxP3^-^T-bet^-^ TILs decreased but still accounted for 55% of total CD4^+^ TILs in D21 tumours, while T_H_1 (FoxP3^-^T-bet^+^) and T_REG_ (FoxP3^+^T-bet^-^) subsets remained relatively stable (**Figure 7A**). Given the high proportion of CD4^+^ TILs exhibiting an effector memory phenotype (CD44^HI^CD62L^-^) in intermediate and late-stage tumours (D14 & D21; **Figure 6A**), these data suggest that as MC38 tumours progress, they are infiltrated by CD4^+^ effector subsets other than T_H_1 and T_REG_ cells, likely T_H_2 or T_H_17 cells. Accompanying these changes in the composition of CD4^+^ TILs, the proportion of CD4^+^ FoxP3^+^T-bet^+^ cells increased during tumour outgrowth (**Figure 7A**).

To gain further insight into the functional status of CD4^+^ TIL subsets present, PD-1 expression in CD4^+^ TILs isolated from late-stage MC38 tumours (D21) was examined (**Figure 7C**). Consistent with an activated or exhausted effector phenotype, a significant proportion of CD4^+^ T_H_1 (FoxP3^-^T-bet^+^) and T_REG_ (FoxP3^+^T-bet^-^ and FoxP3^+^T-bet^+^) cells expressed PD-1 (51%, 25% and 55% PD-1^+^, respectively), whereas most naïve-like cells (FoxP3^-^T-bet^-^) lacked PD-1 expression (84% PD-1^-^; **Figure 7D**). Notably, PD-1 expression was highest in CD4^+^ T_H_1 (FoxP3^-^T-bet^+^, 51% PD-1^+^) and FoxP3^+^T-bet^+^ T_REG_ (55% PD-1^+^) subsets, which may reflect exhaustion (54) and enhanced suppressive function (55, 56) in these subsets, respectively (**Figure 7D**). Altogether, these results indicate that during progressive outgrowth, MC38 tumours are increasingly infiltrated by both anti-tumour T_H_1 effector and suppressive T_REG_ CD4^+^ subsets. However, the polarisation of a large proportion of CD4^+^ TILs (40-75% FoxP3^-^T-bet^-^) cannot be confirmed, suggesting that MC38 tumours are also infiltrated by other T helper subsets not identified here.

### 3.8 Terminally differentiated CD8^+^ effector cell infiltration of MC38 tumours increases during progressive tumour growth

T-bet and Eomes expression within CD8^+^ TILs was also examined to further define their differentiation status. CD8^+^ TILs exhibited distinct single-positive and double-positive T-bet and Eomes populations (**Figures 7B, E**). Similar to observations in CD4^+^ TILs, a large proportion of CD8^+^ TILs in early-stage tumours (D7) were double-negative for both transcription factors (T-bet^-^Eomes^-^, 66% of total CD8^+^ TILs; **Figure 7B**). Again, given the CD44^LO^CD62L^+^ phenotype of a significant proportion of D7 CD8^+^ TILs (**Figure 6B**), these data suggest that MC38 tumours are initially infiltrated by naïve CD8^+^ T cells. During subsequent tumour outgrowth, the frequency of T-bet^-^Eomes^-^ CD8^+^ TILs decreased, while the proportion of effector memory-like (57, 58) T-bet^-^Eomes^+^ and T-bet^+^Eomes^+^ CD8^+^ TILs remained relatively constant (**Figure 7B**). There was also an increase in the proportion of CD8^+^ T-bet^+^Eomes^-^ cells, which composed 30-32% of the total CD8^+^ infiltrate in D14 and D21 MC38 tumours (versus 9% at D7; **Figure 7B**).

To further characterise CD8^+^ TIL populations, the expression of PD-1 in late-stage tumours (D21) was assessed. T-bet^-^Eomes^+^ and T-bet^-^Eomes^-^ CD8^+^ TILs largely lacked PD-1 expression (12% and 18% PD-1^+^, respectively), in accordance with their memory- and naïve- like transcription factor profiles, respectively (**Figures 7E, F**). In contrast, a large proportion of the T-bet^+^Eomes^+^ CD8^+^ subset expressed PD-1 (46%), consistent with the acquisition of an activated or exhausted effector phenotype (**Figure 7F**). PD-1 expression was highest in T-bet^+^Eomes^-^ CD8^+^ TILs, with majority of these cells expressing PD-1 (81%; **Figure 7F**). Given the high expression of T-bet by these cells, this subset likely represents a population of terminally differentiated effectors with a more severe exhausted phenotype (59, 60). Hence, similar to the kinetics of CD4^+^ TIL infiltration, MC38 tumours are increasingly infiltrated by CD8^+^ effectors during progressive tumour outgrowth. However, in later stages of tumour outgrowth, a large proportion of these cells exhibit features of terminal differentiation, particularly the subset distinguished by a T-bet^+^Eomes^-^ phenotype.

### 3.9 CD8^+^ TILs undergo progressive exhaustion during tumour outgrowth and display a severely exhausted phenotype in late-stage MC38 tumours

**Figure 8:**
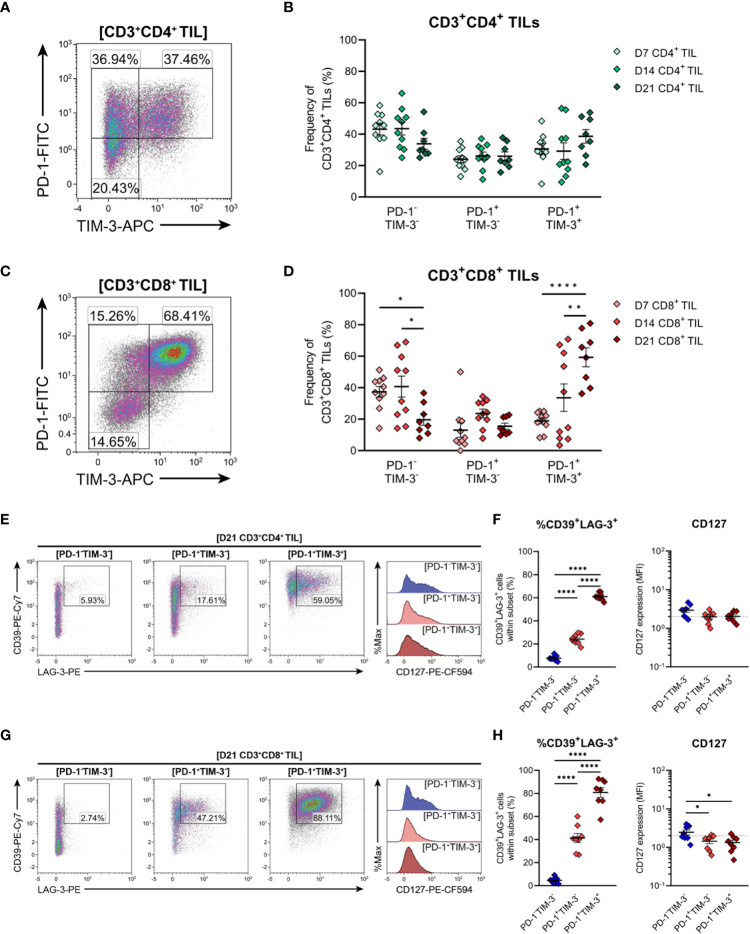
Expression of inhibitory receptors associated with exhaustion by CD4^+^ and CD8^+^ T cells infiltrating MC38 tumours. **(A, C)** Representative density plots depicting the distribution of PD-1 and TIM-3 subsets within total CD3^+^CD4^+^ **(A)** and CD3^+^CD8^+^ **(C)** TILs. **(B, D)** Frequencies of naïve-like (PD-1^-^TIM-3^-^), progenitor exhausted (PD-1^+^TIM-3^-^) and terminally exhausted (PD-1^+^TIM-3^+^) T cells among CD3^+^CD4^+^ **(B)** and CD3^+^CD8^+^ **(D)** TILs. **(E, G)** Representative flow plots depicting LAG-4, CD39 and CD127 expression in naïve-like (PD-1^-^TIM-3^-^; blue), progenitor exhausted (PD-1^+^TIM-3^-^; light red) and terminally exhausted (PD-1^+^TIM-3^+^; dark red) T cell subsets within CD3^+^CD4^+^ **(E)** and CD3^+^CD8^+^ **(G)** TIL isolated from D21 MC38 tumours. **(F, H)** Expression of both CD39 and LAG-3 (%CD39^+^LAG-3^+^) and CD127 (MFI) within PD-1 and TIM-3 subsets of CD3^+^CD4^+^ **(F)** and CD3^+^CD8^+^ **(H)** TIL isolated from D21 MC38 tumours. All density plot percentages indicate frequencies as the proportion of the gated population (indicated in bold titles with square brackets). All data are representative of 8-10 biological replicates per timepoint. Graphs represent the mean and standard error of the mean (SEM, error bars). Statistical analyses were performed using two-way **(B, D)** or one-way **(F, H)** ANOVA with *post-hoc* Tukey test to correct for multiple comparisons. *p ≤ 0.05; **p ≤ 0.01; ****p ≤ 0.0001.

Expression of the inhibitory receptors PD-1 and TIM-3 can be used to classify T cells along a spectrum of dysfunction toward terminal differentiation and exhaustion. At opposing ends of this functional spectrum, PD-1^+^TIM-3^+^ cells are considered severely dysfunctional or ‘terminally exhausted’, whereas PD-1^−^TIM-3^−^ cells represent functional effectors (33, 59, 61). In between these phenotypic extremes, PD1^+^TIM3^−^ cells are thought to represent T cells in a transitional status, termed ‘transitional exhausted’ cells (62). Therefore, to clarify the exhaustion status of MC38 TILs, the distribution of PD-1/TIM-3 subsets within CD4^+^ and CD8^+^ TILs were examined along with the expression of additional exhaustion-associated markers in these subsets. The composition of PD-1 and TIM-3 subsets within CD4^+^ TILs remained relatively stable throughout the course of MC38 tumour progression, with distinct PD-1^-^TIM-3^-^ (34-44%), PD-1^+^TIM-3^-^ (24-26%) and PD-1^+^TIM-3^+^ (29-39%) populations apparent (**Figures 8A, B**). In contrast to CD4^+^ TILs, the composition of PD-1 and TIM-3 subsets within CD8^+^ TILs changed over the course of tumour outgrowth (**Figures 8C, D**). In early-stage tumours (D7), CD8^+^ TILs were predominantly PD-1^-^TIM-3^-^, but as tumours progressed, there was a decrease in the proportion of PD-1^-^TIM-3^-^ cells (37→20%) and a concomitant increase in the proportion of terminally exhausted PD-1^+^TIM-3^+^ cells (19→59%; **Figure 8D**). Meanwhile, the proportion of transitional exhausted PD-1^+^TIM-3^-^ cells remained relatively stable (**Figure 8D**). Consistent with progressive T cell exhaustion, the simultaneous expression of additional inhibitory receptors – specifically LAG-3 and CD39 – increased across PD-1 and TIM-3 subsets in both CD4^+^ and CD8^+^ TILs in a hierarchical manner (%CD39^+^LAG-3^+^; PD-1^-^TIM-3^-^<PD-1^+^TIM-3^-^<PD-1^+^TIM-3^+^; **Figures 8E–H**). Notably, the majority of PD-1^+^TIM-3^+^ cells co-expressed CD39 and LAG-3 (61% and 81% for CD4^+^ and CD8^+^ subsets, respectively; **Figures 8F, H**). Furthermore, CD8^+^ TILs exhibited hierarchical downregulation of CD127 across PD-1 and TIM-3 subsets, wherein the terminally exhausted PD-1^+^TIM-3^+^ subset lacked CD127 expression – a hallmark of terminal differentiation (63) (**Figure 8H**). Altogether, these data suggest that CD8^+^ TILs undergo progressive exhaustion during the outgrowth of MC38 tumours.

### 3.10 Tumour-infiltrating CD4^+^ and CD8^+^ T cells that express PD-1 are polyfunctional and display rapid effector functions *ex vivo*

**Figure 9:**
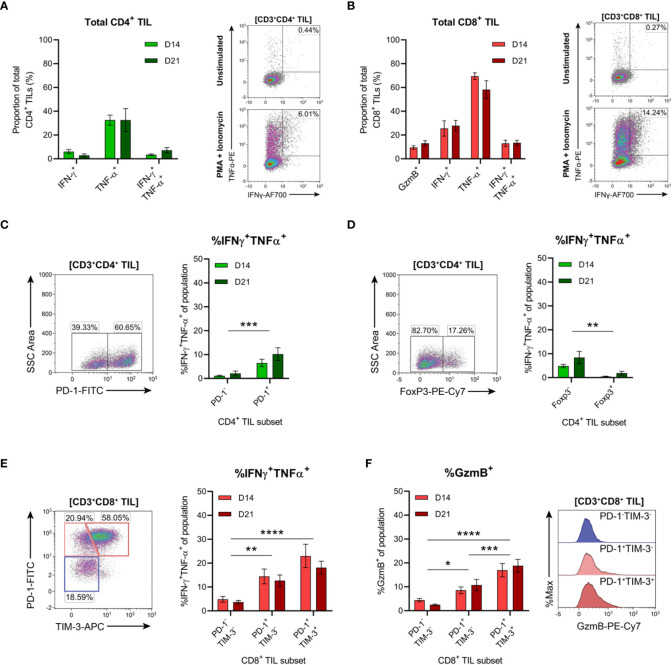
Tumour-infiltrating CD4^+^ and CD8^+^ T cell subsets exhibit distinct functional properties. **(A, B)** Frequency of cytokine producing cells within total CD3^+^CD4^+^ **(A)** and CD3^+^CD8^+^ **(B)** TILs. Representative density plots depict IFN-γ and TNF-α production by total CD3^+^CD4^+^ and CD3^+^CD8^+^ TILs in unstimulated controls (top) and following PMA+ionomycin stimulation (bottom). **(C, D)** Representative density plots depicting PD-1 **(C)** and FoxP3 **(D)** subset gating in CD3^+^CD4^+^ TILs and frequencies of cells producing both IFN-γ and TNF-α (%IFN-γ^+^TNF-α^+^) within PD-1 and FoxP3 subsets. **(E)** Representative density plot depicting PD-1 and TIM-3 subset gating in CD3^+^CD8^+^ TILs and frequencies of cells producing both IFN-γ and TNF-α (%IFN-γ^+^TNF-α^+^) within these subsets. **(F)** Frequency of cells producing granzyme B (%GzmB^+^) within PD-1 and TIM-3 subsets (CD3^+^CD8^+^ TILs) and representative histogram overlays. All density plot percentages indicate frequencies as the proportion of the gated population (indicated in bold titles with square brackets). Graphs represent the mean and standard error of the mean (SEM, error bars). Statistical analyses were performed using two-way ANOVA with *post-hoc* Tukey test to correct for multiple comparisons, or by multiple unpaired Student’s *t*-tests, using the Holm-Sidak method to correct for multiple comparisons. *p ≤ 0.05; **p ≤ 0.01; ***p ≤ 0.001; ****p ≤ 0.0001.

The functional qualities of CD4^+^ and CD8^+^ T cells infiltrating D14 and D21 tumours were examined *ex vivo* following tumour dissociation. Effector function was assessed by intracellular cytokine staining following acute PMA and ionomycin stimulation and in unstimulated controls. Following stimulation, CD4^+^ TILs mainly produced TNF, with low frequencies of cells producing IFN-γ or both cytokines (<10%; **Figure 9A**). Compared to CD4^+^ TILs, a greater proportion of CD8^+^ TILs produced IFN-γ and were polyfunctional, producing both IFN-γ and TNF (13% versus 5%; **Figure 9B**). A similar proportion of CD8^+^ TILs also expressed granzyme B (9-13% GzmB^+^; **Figure 9B**). However, production of IL-2 or IL-10 in either CD4^+^ or CD8^+^ TILs could not be detected (data not shown) and unstimulated controls did not exhibit significant frequencies of cytokine-producing cells (~1%; **Figures 9A, B**, representative density plots). In response to stimulation, there were no significant differences in the frequencies of cytokine-producing CD4^+^ or CD8^+^ cells between D14 and D21 tumours (%IFN-γ^+^, %TNF^+^, %IFN-γ^+^TNF^+^ and %GzmB^+^; **Figures 9A, B**).

In addition to the progressive upregulation of inhibitory receptors (particularly PD-1), T cell exhaustion is typically characterised by the stepwise loss of effector functions such as cytokine polyfunctionality and lytic activity (64, 65). Therefore, we investigated whether PD-1 expression was associated with the loss of effector functions within CD4^+^ and CD8^+^ TILs. Co-production of IFN-γ and TNF was analysed within CD4^+^ TIL subsets based on the presence or absence of PD-1 or FoxP3 expression (**Figures 9C, D**). Polyfunctional CD4^+^ TILs (IFN-γ^+^TNF^+^) were enriched within cells lacking FoxP3 expression (compared to FoxP3^+^ counterparts) and within PD-1^+^ cells (compared to PD-1^-^ counterparts; **Figures 9C, D**). There was no difference in the proportion of polyfunctional PD-1^+^ CD4^+^ TILs between D14 and D21 tumours, suggesting that their functionality did not decline during progressive tumour growth (**Figure 9C**). These data support the notion that FoxP3^+^ CD4^+^ TILs represent subsets of T_REG_ cells, while CD4^+^ T_H_1 effectors are enriched in the PD-1^+^ TIL fraction.

In CD8^+^ TILs, IFN-γ and TNF co-production along with granzyme B expression was assessed within PD-1 and TIM-3 subsets (**Figures 9E, F**). Despite their exhausted phenotype, a greater proportion of PD-1^+^TIM-3^+^ CD8^+^ TILs produced both IFN-γ and TNF and displayed increased cytolytic potential (with a higher frequency of granzyme B-positive cells) compared to the PD-1^+^TIM-3^-^ and PD-1^-^TIM-3^-^ subsets (**Figures 9E, F**). Within all CD8^+^ TIL subsets, there were no differences in the frequencies of polyfunctional or granzyme B-expressing cells between D14 and D21 tumours, suggesting that these subsets also maintain their functional status during tumour progression (**Figures 9E, F**). Together, these data indicate that despite their exhausted phenotype, PD-1^+^TIM-3^+^ CD8^+^ TILs retain rapid effector functions *ex vivo* and display enhanced polyfunctionality compared to other CD8^+^ TIL subsets. Furthermore, both CD4^+^ and CD8^+^ T cells with anti-tumour functionality (particularly IFN-γ and TNF co-production) are enriched within the PD-1^+^ fraction of MC38 TILs.

### 3.11 Endogenous CD8^+^ T cell responses to MC38 are dominated by recognition of the p15E tumour antigen over Adpgk and Dpagt1 neoantigens

Three MHC-I-restricted neoantigens have been reported in the MC38 colon adenocarcinoma cell line (66). These neoantigens derive from non-synonymous point-mutations in the genes encoding ADP-dependent glucokinase (Adpgk), dolichyl-phosphate N-acetylglucosaminephosphotransferase 1 (Dpagt1) and RalBP1-associated Eps domain-containing 1 (Reps1) (66). To validate these neoantigens as potential targets for endogenous CD8^+^ T cell responses, the presence of these mutations was assessed in our MC38 cell line. Two mutated neoantigens (Adpgk_R304M_ and Dpagt1_V304L_) were detected, while the C→G point mutation reported in *Reps1* (Reps1_P45A_) was absent in our version of the MC38 cell line (**Supplementary Figure 5**).

**Figure 10:**
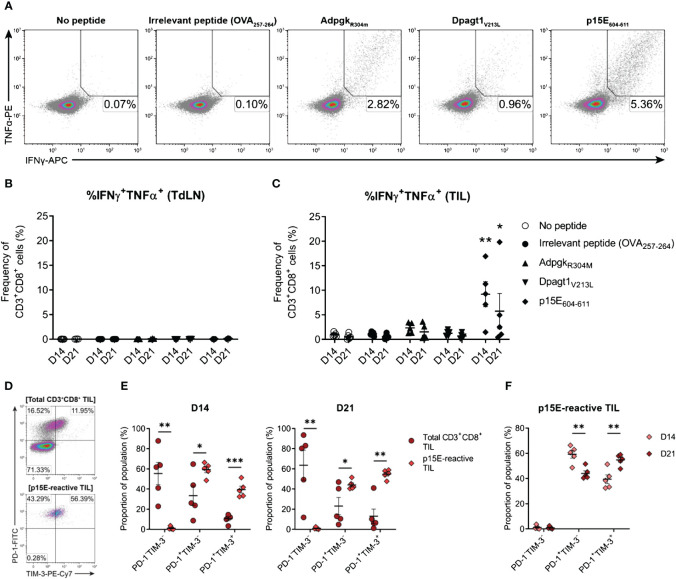
The endogenous CD8^+^ T cell response to MC38 is dominated by recognition of the p15E tumour antigen. **(A)** Representative density plots showing TNFα and IFNγ production by CD8^+^ TILs stimulated *ex vivo* with BMDCs pulsed with 1 µg/mL of H-2K^b^/D^b^-restricted tumour antigen peptides (p15E_604-611_, KSPWFTTL; Adpgk_R304M,_ ASMTNMELM; and Dpagt1_V213L_, SIIVFNLL), unpulsed BMDCs (no peptide) or BMDC pulsed with 1 µg/mL OVA_257–264_ peptide (SIINFEKL; irrelevant peptide). Percentages indicate peptide-reactive CD8^+^ T cell frequencies (%IFNγ^+^TNFα^+^) as the proportion of total CD3^+^CD8^+^ cells. **(B, C)** Frequency of polyfunctional CD3^+^CD8^+^ cells – isolated from TdLNs **(B)** and TILs **(C)** – producing IFNγ and TNFα in response to indicated treatments (%IFNγ^+^TNFα^+^). **(D)** Representative density plots showing PD-1 and TIM-3 expression by total CD3^+^CD8^+^ TILs (top) and p15E-reactive CD3^+^CD8^+^ TILs (IFNγ^+^TNFα^+^, bottom) isolated from D21 MC38 tumours. Percentages indicate subset frequencies as the proportion of the gated populations. **(E)** Comparison of PD-1 and TIM-3 subset compositions in total CD3^+^CD8^+^ TILs (•) and p15E-reactive CD3^+^CD8^+^ TILs (♦) from D14 and D21 MC38 tumours. **(F)** Comparison of PD-1 and TIM-3 subset compositions in p15E-reactive CD3^+^CD8^+^ TILs from D14 and D21 MC38 tumours. All data are representative of five biological replicates per timepoint. Graphs represent the mean (line) and standard error of the mean (SEM, error bars). Statistical analyses were performed using two-way ANOVA with *post-hoc* Dunnett’s test, comparing tumour antigen peptide-reactive frequencies to irrelevant peptide controls **(B, C)**, or multiple unpaired Student’s t-tests using the Holm-Sidak method to correct for multiple comparisons **(E, F)**. *p ≤ 0.05; **p ≤ 0.01; ***p ≤ 0.001.

Having confirmed that MC38 cells harboured mutations in *Adpgk* and *Dpagt1*, the recognition of neo-epitopes derived from these mutations by endogenous CD8^+^ T cells was assessed (Adpgk_R304M_, ASMTNMELM; Dpagt1_V213L_, SIIVFNLL). In addition, CD8^+^ T cell responses to p15E (p15E_604–611_, KSPWFTTL) – an envelope protein of a murine endogenous retrovirus (MERV) and an immunodominant tumour antigen expressed by the MC38 cell line (67, 68) – was also examined. CD3^+^CD8^+^ T cells were isolated from D14 and D21 tumour-bearing mice and stimulated *ex vivo* with peptide-pulsed bone marrow-derived dendritic cells (BMDCs). Antigen-specific CD8^+^ T cell responses were then evaluated by intracellular cytokine staining for IFN-γ and TNF (**Figure 10A**). No antigen-specific activation of CD3^+^CD8^+^ T cells isolated from tumour-draining lymph nodes (TdLN) could be detected (**Figure 10B**). However, CD8^+^ TILs strongly responded to p15E_604–611_, with p15E-reactive CD8^+^ TILs readily detectable in D14 and D21 tumours (**Figure 10C**). Polyfunctional responses against the Adpgk_R304M_ neo-epitope could be detected in some mice, although to a lesser magnitude than p15E, composing up to 3.3% and 3.6% of total CD3^+^CD8^+^ in D14 and D21 tumours, respectively (**Figure 10C**). Although Dpagt1_V213L_-reactive CD8^+^ TILs were detectable in some animals, this neo-epitope did not elicit strong activation of CD8^+^ TILs from either D14 or D21 tumours (**Figure 10C**). These data suggest that the CD8^+^ T cell response against MC38 tumours is dominated by recognition of the endogenous retroviral envelope protein, p15E, over the identified neoantigens, Adpgk_R304M_ and Dpagt1_V213L_. Importantly, CD8^+^ T cell responses against p15E and Adpgk_R304M_ occur spontaneously in MC38 tumour-bearing mice, making these tumour antigens relevant immunotherapeutic targets in this model.

### 3.12 p15E-reactive CD8^+^ TILs display phenotypic features of exhaustion which exacerbate during tumour progression

After confirming the presence of functional tumour-specific CD8^+^ T cells in MC38 tumours, their phenotypic status was examined – specifically, the expression of inhibitory receptors by p15E-reactive CD8^+^ TILs. Bulk CD8^+^ TILs from D14 and D21 tumours were heterogeneous in their expression of both PD-1 and TIM-3, while virtually all p15E-reactive CD8^+^ TILs were PD-1^+^ (>99% in both D14 and D21 tumours) with a subset of these cells co-expressing TIM-3 (**Figures 10D, E**). Hence, despite their functional status *ex vivo* (IFN-γ and TNF production), these tumour-specific CD8^+^ T cells exhibited phenotypic features of exhaustion, containing populations of both transitional exhausted (PD-1^+^TIM-3^-^) and terminally exhausted (PD-1^+^TIM-3^+^) cells. Further analysis of p15E-reactive TILs revealed that the proportion of terminally exhausted PD-1^+^TIM-3^+^ cells increased during tumour progression, with a concomitant decrease in the proportion of transitional exhausted PD-1^+^TIM-3^-^ cells (D21 versus D14 tumours; **Figure 10F**). These data demonstrate that tumour-specific T cells within MC38 tumours acquire the expression of inhibitory receptors in a stepwise manner during tumour progression.

## 4 Discussion

Immune-competent murine tumour models are crucial tools for the development and optimisation of cancer immunotherapies. However, understanding how these models recapitulate the biology of human disease is vital for the translation of preclinical findings into the clinical setting. In this study, the landscape of T cell infiltration in MC38 tumours was systematically examined, with the aim of determining how this model reflects features of human CRC.

**Figure 11:**
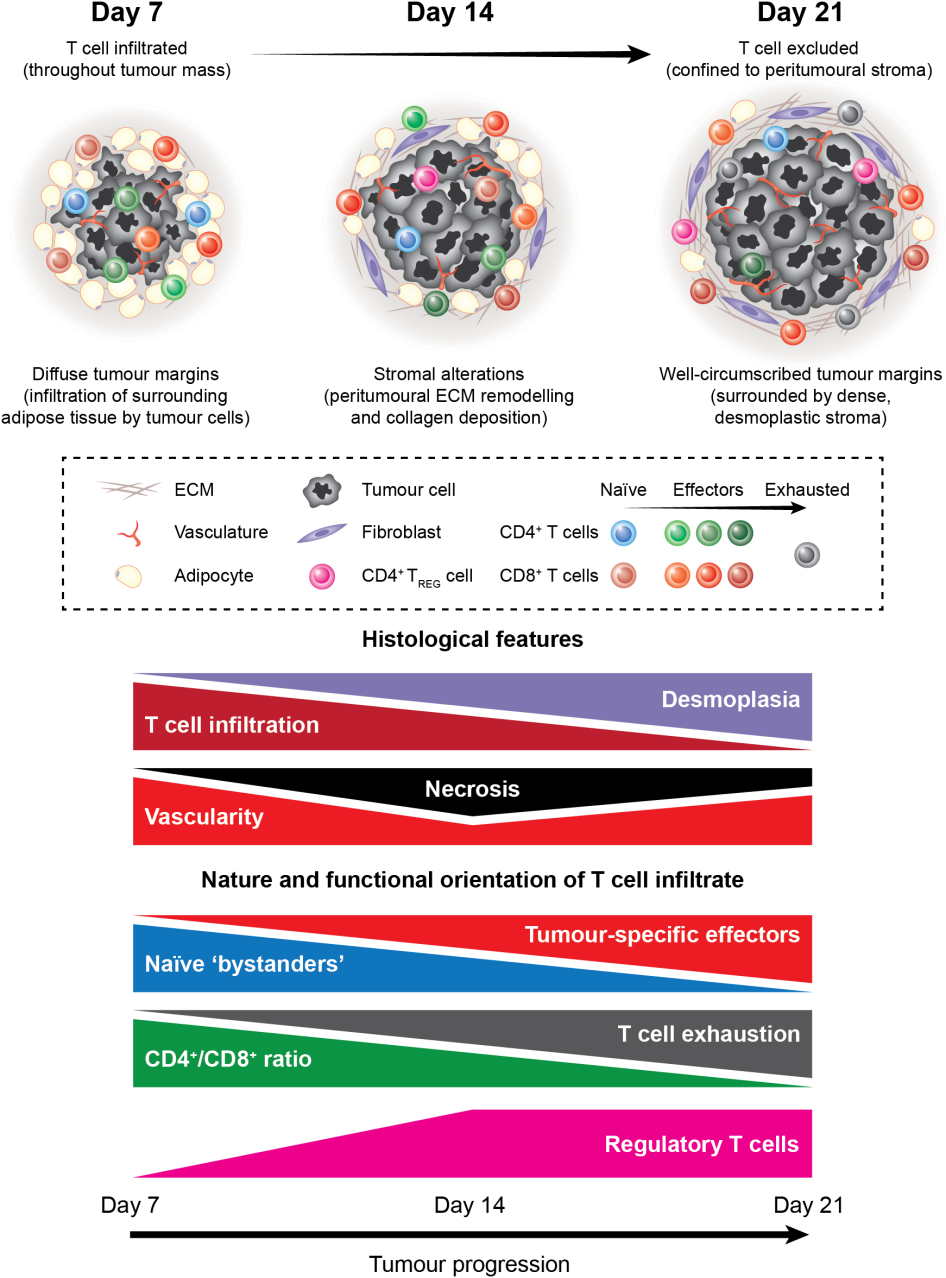
The tumour-T cell immune landscape in MC38 tumours during progressive outgrowth. Extensive characterisation of outgrowing MC38 tumours revealed dynamic histological changes and shifts in the nature and functional orientation of infiltrating T cells. Histological changes included stromal alterations, particularly peritumoural ECM deposition and remodelling that resulted in the formation of dense desmoplastic stroma at the tumour margin. This coincided with decreasing T cell infiltration into the tumour core, with late-stage MC38 tumours exhibiting a T cell-excluded phenotype. In addition, necrosis, angiogenesis, and vascular abnormalities were apparent within the MC38 tumour microenvironment. Longitudinal immunophenotyping of TILs in MC38 tumours revealed dynamic changes in the composition, phenotype, and function of these cells. Notably, the T cell infiltrate in MC38 tumours shifted from cells with a naïve-like phenotype toward a more pro-inflammatory composition during progressive outgrowth. This was characterised by an increase in CD8^+^ effectors and a decrease in the CD4^+^/CD8^+^ ratio within MC38 tumours. In parallel, CD8^+^ TILs progressively acquired phenotypic features of dysfunction, which cumulated in a profoundly exhausted phenotype in late-stage MC38 tumours. Our data also indicate that unfavourable immune populations such as suppressive CD4^+^ regulatory T cells also accumulate in outgrowing MC38 tumours.

We present an overview of the tumour-T cell immune landscape in subcutaneous MC38 tumours and how the MC38 TME evolves over the course of tumour progression (**Figure 11**). Early MC38 tumours (D7) exhibit a nascent TME, with perfuse infiltration throughout the tumour mass by T cells that predominately display naïve and early effector phenotypes. Subsequently, T cell infiltration is increasingly restricted from the tumour core, which coincides with the accumulation of fibrotic, desmoplastic stroma and the deposition of dense reticular fibres at the invasive margin. Intermediate MC38 tumours (D14) begin to acquire features of a mature TME – with ECM remodelling and a shift in the composition and phenotype of infiltrating T cells towards effector populations – while late-stage tumours (D21) exhibit a mature TME reminiscent of human tumours, with desmoplasia, CD8^+^ T cell exhaustion, and T cell exclusion from the tumour core. MC38 tumours also display extensive vascularisation throughout the tumour mass. However, while T cell extravasation is permitted by vessels in adjacent connective tissue and more structurally ‘normal’ vessels at the periphery of tumours, T cells are unable to access the tumour interior *via* intratumoural vasculature. Hence, MC38 tumour progression is characterised by the development of an T cell-excluded phenotype, which appears to be mediated by physical barriers, particularly dense ECM and aberrant tumour vasculature.

Relevant to these changes, we observed that MC38 tumour cells secreted TGF-β *in vitro* and that high levels of this cytokine were present within the MC38 TME. Notably, TGF-β plays a central role in the recruitment of fibroblasts to tumours and their conversion to cancer-associated fibroblasts, while increased TGF-β expression correlates with the accumulation of fibrotic desmoplastic stroma (69) and is strongly associated with T cell exclusion in human tumours (17, 70, 71). Thus, high TGF-β concentrations within the MC38 TME – coinciding with the development of desmoplastic stroma and T cell exclusion at the tumour margin – may imply a role for this cytokine in the induction of this phenotype. Consistent with this notion, others have identified TGF-β as a mechanism of immune evasion employed by MC38 tumours, acting to impair anti-tumour T cell function while promoting T cell exclusion (17).

In the context of human CRC, high levels of TGF-β and ECM remodelling are defining features of CMS4 tumours (mesenchymal subtype), which exhibit the poorest prognosis across all CRC subtypes (22). Akin to our observations in MC38 tumours, CMS4 tumours frequently display an immune-excluded phenotype with prominent desmoplasia and are dependent on a TGF-β-driven stromal gene program, in which cancer-associated fibroblasts promote tumour progression (72–74). However, CMS4 tumours are predominately microsatellite stable (MSS) and exhibit high chromosomal instability (CIN), whereas the MC38 model is hypermutated and exhibits high microsatellite instability (MSI-H) which are defining characteristics of human CMS1 tumours (MSI immune subtype) (19, 22). Despite this disparity between the genomic features of human CMS4 tumours and MC38, our findings show that late-stage MC38 tumours recapitulate archetypal features of the TME in human CMS4 tumours. This study therefore supports the use of MC38 as a relevant model for the immune-excluded phenotype observed in CMS4 tumours and for investigating therapeutic strategies that aim to reverse T cell exclusion.

Longitudinal immunophenotyping of TILs over the course of MC38 tumour progression revealed dynamic changes in the composition, phenotype, and function of these cells (**Figure 11**). Notably, decreases in the ratios of CD4^+^/CD8^+^, CD4^+^FoxP3^+^/T-bet^+^CD8^+^ and CD4^+^T-bet^+^/CD4^+^FoxP3^+^ TILs were observed during progressive tumour outgrowth, suggesting that the T cell infiltrate in MC38 tumours shifts toward a more pro-inflammatory composition during progressive outgrowth. However, at the same time, T cells are increasingly excluded from the vicinity of tumour cells. Immune exclusion – as well as other immunosuppressive mechanisms – may therefore explain the unabated growth of these tumours despite a strong endogenous anti-tumour T cell response.

While pronounced T cell infiltration in tumours is generally considered a manifestation of an ongoing host immune response against malignant cells, the mere presence of TILs is not necessarily indicative of an effective anti-tumour immune response. Rather, the ability of TILs to mediate tumour regression relies on both the quantity and the quality of responding T cell infiltrates (75, 76). Consequently, the phenotypes of T cells isolated from MC38 tumours were examined, revealing striking alterations over the course of tumour progression. Regarding CD8^+^ T cells, infiltration by CD8^+^ effectors increased during outgrowth of MC38 tumours, but these cells progressively acquired phenotypic features of exhaustion. Interestingly, PD-1 and CD69 were co-expressed within a large proportion of CD8^+^ TILs, which may imply these cells are tissue-resident memory cells (T_RM_ cells) (77, 78), although further phenotypic analysis is required to confirm this phenotype. However, as T_RM_ formation is strictly dependent on TGF-β (79, 80), it is plausible that abundant TGF-β within the MC38 TME drives CD8^+^ T_RM_ differentiation, while also mediating the exclusion of these cells from the tumour parenchyma (71).

In late-stage MC38 tumours, CD8^+^ TILs displayed a profoundly exhausted phenotype characterised by simultaneous expression of multiple inhibitory receptors (PD-1, TIM-3, LAG-3 and CD39) and low expression of CD127. In addition, an increasing proportion of CD8^+^ TILs exhibited high T-bet expression and low expression of Eomes as tumours progressed. Given the association of high T-bet expression with the acquisition of effector function (81, 82), and the requirement for Eomes expression in memory formation (57, 58), these CD8^+^ T-bet^+^Eomes^-^ TILs likely represent dysfunctional, terminally differentiated effectors. We also observed that tumour-specific CD8^+^ TILs recognising the p15E tumour antigen displayed phenotypic features of exhaustion and that the proportion of these cells displaying a terminally exhausted PD-1^+^TIM-3^+^ phenotype increased with tumour progression. Interestingly, in contradiction to their dysfunctional phenotype, these cells were capable of rapid IFN-γ and TNF production *ex vivo*, while CD8^+^ TILs with anti-tumour functionality *ex vivo* (IFN-γ and TNF co-production) were predominately PD-1^+^. While these data imply that these cells are not exhausted, it more likely reflects their highly differentiated state skewed toward potent effector function when simulated *ex vivo*. By contrast, these cells would be highly vulnerable to suppression within the TME given their expression of multiple inhibitory receptors (PD-1, TIM-3, LAG-3 and CD39), which could render them dysfunctional and unable mediate effective tumour control *in situ*. Although the factors driving the exhaustion of CD8^+^ T cells in MC38 tumours is unclear, chronic tumour antigen stimulation (83) and/or suppressive factors within the tumour microenvironment (65) are likely involved. Indeed, PD-L1 expression by both MC38 tumour cells and tumour-infiltrating myeloid cells has been shown to suppress of anti-tumour CD8^+^ T cell responses in this model (15, 84). Consequently, this study supports the use of the MC38 model in studies investigating immunotherapies targeting T cell exhaustion and the PD-1/PD-L1 axis and as a model for microsatellite instable-high (MSI-H) human CRCs that exhibit high PD-L1 expression and responsiveness to PD-1 blockade (28, 85).

While CD8^+^ TILs isolated from MC38 tumours displayed well-defined phenotypes, CD4^+^ TILs were highly heterogenous and difficult to define in this study. Indeed, CD4^+^ T cell polarisation and function is extremely diverse, requiring more comprehensive phenotyping than that used in this study to delineate subpopulations. Notably, the majority of CD4^+^ TILs in MC38 tumours did not express FoxP3 or T-bet (40-75%), suggesting that MC38 tumours are heavily infiltrated by effector subsets other than T_H_1 and T_REG_ cells, likely T_H_2 or T_H_17 cells. Nevertheless, tumour progression was accompanied by increased infiltration of activated effector memory CD4^+^ T cells and T_H_1 (FoxP3^-^T-bet^+^) and T_REG_ (FoxP3^+^T-bet^-^) subsets were present in established tumours. We also observed a proportion of CD4^+^ TILs with a FoxP3^+^T-bet^+^ phenotype that increased during tumour outgrowth. Interestingly, CD4^+^ T cells exhibiting this phenotype have been identified as a subset of highly suppressive T_REG_ cells which accumulate at sites of type-1 inflammation and potently inhibit local T_H_1 immune responses (86–88). CD4^+^ TIL subpopulations also exhibited CD25^+^CD137^+^ and CD39^+^TIM-3^+^ phenotypes (data not shown), supporting the notion that CD4^+^ T_REG_ cells accumulate in MC38 tumours given that these markers are highly and selectively expressed by this subset (89–92). In established tumours, CD4^+^ T_H_1 TILs displayed high PD-1 expression, which was also associated with polyfunctional IFN-γ and TNF production *ex vivo*. Altogether, these data suggest that recruitment of T_REG_ cells is an active immunosuppressive mechanism in the MC38 model and that CD4^+^ T_H_1 effectors may undergo a similar fate to CD8^+^ TILs, acquiring an exhausted phenotype within the MC38 TME.

Aside from the phenotypic features of T cell infiltration in MC38 tumours, this study also characterised the frequency of CD8^+^ TILs responsive to peptide stimulation with tumour antigens (TAs) identified in the MC38 model. In an interesting observation, detectable tumour-specific CD8^+^ T cell responses were dominated by recognition of the p15E ERV antigen over two defined mutated neoantigens, with functional p15E-reactive clones composing up to ~20% of total CD8^+^ TILs in late-stage tumours. Although mutated neoantigens have generated intense interest as immunotherapy targets (93), TAs derived from endogenous retroviral elements are increasingly promising alternatives (94). Unlike neoantigens – which derive from mutations and are often unique to individual patients – ERV-derived TAs arise through epigenetic dysregulation of the cancer genome and are shared across different patients and tumour types (95, 96). Consequently, this class of TAs is amenable to the development of off-the-shelf therapies for patients with shared HLA class I alleles. MC38 and the p15E tumour antigen may therefore represent a useful preclinical model for developing immunotherapies that target shared ERV TAs.

One limitation of this study is the use of peptide stimulation, which can only detect responsive or ‘reactive’ T cells and is therefore prone to underestimating the true frequency of antigen-specific T cells. Indeed, other studies using more reliable peptide-MHC tetramer staining have reported higher frequencies tumour-specific T cells in the MC38 model, with Dpagt1-specific CD8^+^ T cells composing over 20% of CD8^+^ TILs in D13 tumours (97). A recent study also identified another neoantigen in the MC38 model derived from a mutation in the ribosomal protein L18 (Rpl18) (98). Strikingly, recognition of this neo-epitope was shown to dominate endogenous CD8^+^ T cell responses compared to other identified neoantigens, including Adpgk_R304M_ (98). Future studies utilising peptide-MHC tetramers should comprehensively examine the immunodominance of defined TAs in the MC38 model, including ERV-derived TAs, mutated neoantigens, and unaltered self-proteins that are overexpressed (such as p53 (99), survivin (100) and topoisomerase II-α (101)). Another question that remains to be addressed regards the identity of MHC class II-restricted tumour antigens recognised by CD4^+^ T cells in the MC38 model. Indeed, there is increasing interest in targeting MHC class II-restricted antigens in immunotherapies (102–104) and mutated neoantigens that stimulate anti-tumour CD4^+^ T cell responses have recently been identified in other syngeneic tumour models (103, 105, 106). A deeper understanding of the immunodominance of different TA classes will help to inform the development of effective immunotherapies.

In human patients with clinically-manifest or advanced disease, tumours have been shaped by years – if not decades – of tumour-immune interactions (107) and are frequently resistant to immunotherapeutic interventions (108). By contrast, tumour-immune interactions in preclinical mouse models occur over a timeframe of days to weeks and studies typically begin therapeutic inventions only days after tumour inoculation. Consequently, how accurately these models recapitulate tumour-immune interactions in humans and the selection of appropriate timepoints in these studies are issues frequently overlooked.

Our observations have important implications for optimal timepoint selection in the MC38 model. Many seminal immunotherapy studies that provide rational for subsequent clinical trials initiate treatment 5-9 days post-inoculation when MC38 tumours are newly established (17, 30, 31, 84, 109–112). However, at these timepoints, we show that MC38 tumours exhibit a nascent TME which lacks both important hallmarks of human tumours and pre-empts the manifestation of the immune-resistance mechanisms that these therapies target (such as T cell exhaustion and immune exclusion). Consequently, at early timepoints, the ability of these immunotherapies to induce tumour regression likely reflects their efficacy before immune-resistant mechanisms in the MC38 TME are established, rather than their ability to reverse these mechanisms or permit anti-tumour immunity in the context of a mature TME. Indeed, antibodies targeting CTLA-4 or TIM-3 have been shown to be effective in various mouse tumour models when administered early (day 3 post-inoculation) but are largely ineffective against established tumours (day 11 post-inoculation) (113). Premature timepoint selection in preclinical tumour models may therefore contribute to disparities between the results of these studies and subsequent clinical trials.

The application of more relevant timepoints (when tumours more accurately reflect the TME in human tumours) may improve the clinical translation of immunotherapies developed in preclinical models such as MC38. Our findings demonstrate that MC38 tumours more faithfully resemble human tumours after 14 days and advocate for the initiation of therapeutic interventions at this timepoint in studies investigating immunotherapies. However, one important caveat concerns balancing the use of more appropriate timepoints with the ethical use of laboratory animals. Although more relevant, the implementation of later timepoints in murine tumour studies may encroach on humane endpoints (based on tumour size) and impair animal welfare. This is a vital consideration, as researchers have a responsibility to perform studies to the highest quality whilst also ensuring the ethical and humane care and use of animals. Ultimately, more accurate application of preclinical models will improve clinical translation and, in accordance with the 3Rs (Replacement, Reduction and Refinement), aid in reducing animal usage.

To conclude, this study describes the dynamics of endogenous T cell responses in the MC38 model, including the spatiotemporal features, composition, phenotype, function, and nature of tumour antigen recognition. Future work should characterise the broader immune context of the MC38 TME, particularly the tumour-infiltrating myeloid compartment given the important role of these cells in tumour immunity and the MC38 model (15, 114). We also identify immune-resistance mechanisms in this model that recapitulate aspects of human CRC subtypes and highlight the importance of appropriate timepoint selection when investigating features of the TME – particularly T cell exclusion and exhaustion. Although cancer immunotherapies hold immense promise, human tumours are heterogeneous and utilise multiple resistance mechanisms that restrain anti-tumour immunity. Therefore, targeting the specific mechanisms active in any given tumour is likely a prerequisite for therapeutic response. Accordingly, personalised combination strategies that are tailored to individual patients’ specific TMEs hold immense promise for the next generation of cancer immunotherapies (115). However, the development of rational combination strategies will be dependent on the appropriate application of preclinical models matched to distinct tumour-immune profiles seen in human patients. Collectively, our findings provide a valuable resource that enables appropriate application of the MC38 model in preclinical studies and will aid in the development and clinical translation of new immunotherapies.

## Data availability statement

The original contributions presented in this study are included in the article and **Supplementary Material**. Further inquiries can be directed to the corresponding author.

## Ethics statement

The animal study was reviewed and approved by University of Otago Animal Ethics Committee.

## Author contributions

NS was the primary designer, performer, and interpreter of experiments, and writer of this manuscript. EP and SN assisted with experimental work, the interpretation of data, and writing the manuscript. AF and MS provided supervision, interpreted results, and reviewed and edited the manuscript. SY was the principal investigator responsible for supervision, conceived the study, provided funding and resources, and reviewed and edited the manuscript. All authors contributed to the article and approved the submitted version.
