# Corrigendum: Late-stage MC38 tumours recapitulate features of human colorectal cancer – implications for appropriate timepoint selection in preclinical studies

**DOI:** 10.3389/fimmu.2024.1430460

**Published:** 2024-06-11

**Authors:** Nicholas J. Shields, Estelle M. Peyroux, Angela L. Ferguson, Megan Steain, Silke Neumann, Sarah L. Young

**Affiliations:** ^1^ School of Medical Sciences, Faculty of Medicine and Health, The University of Sydney, Sydney, NSW, Australia; ^2^ Department of Pathology, Otago Medical School, University of Otago, Dunedin, New Zealand; ^3^ Liver Injury and Cancer Program, Centenary Institute, Sydney, NSW, Australia; ^4^ Charles Perkins Centre, University of Sydney, Sydney, NSW, Australia; ^5^ Faculty of Science, University of Canterbury, Christchurch, New Zealand

**Keywords:** colorectal cancer, immunotherapy, syngeneic preclinical models, MC38, immune exclusion, t cell exhaustion, tumour microenvironment

In the published article, there was an error in **Figure 10** (panel D) as published. The representative density plot labelled “p15E-reactive TIL” is incorrect and has been mistakenly duplicated from the plot above labelled “Total CD3^+^CD8^+^ TIL”. The existing caption for this Figure is correct as published. The corrected **Figure 10** appears below:

**Figure 10:**
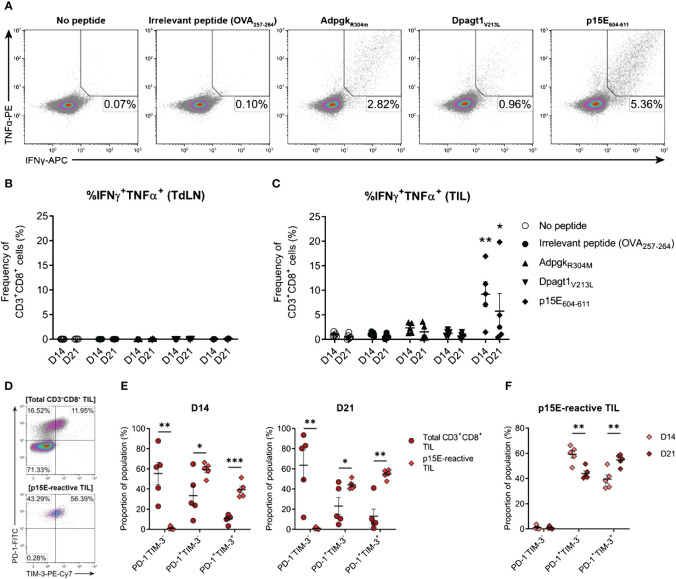
The endogenous CD8 T cell response to MC38 is dominated by recognition of the p15E tumour antigen. **(A)** Representative density plots showing TNFα and IFNγ production by CD8^+^ TILs stimulated *ex vivo* with BMDCs pulsed with 1 µg/mL of H-2K^b^/D^b^-restricted tumour antigen peptides (p15E_604-611_, KSPWFTTL; Adpgk_R304M_, ASMTNMELM; and Dpagt1_V213L_, SIIVFNLL), unpulsed BMDCs (no peptide) or BMDC pulsed with 1 µg/mL OVA_257 -264_ peptide (SIINFEKL; irrelevant peptide). Percentages indicate peptide-reactive CD8^+^ T cell frequencies (%IFNγ^+^TNFα^+^) as the proportion of total CD3^+^CD8^+^ cells. **(B, C)** Frequency of polyfunctional CD3^+^CD8^+^ cells - isolated from TdLNs **(B)** and TILs **(C)** - producing IFNγ and TNFα in response to indicated treatments (%IFNγ^+^TNFα^+^). **(D)** Representative density plots showing PD-1 and TIM-3 expression by total CD3^+^CD8^+^ TILs (top) and p15E-reactive CD3^+^CD8^+^ TILs (IFNγ^+^TNFα^+^, bottom) isolated from D21 MC38 tumours. Percentages indicate subset frequencies as the proportion of the gated populations. **(E)** Comparison of PD-1 and TIM-3 subset compositions in total CD3^+^CD8^+^ TILs (•) and p15E-reactive CD3^+^CD8^+^ TILs (♦) from D14 and D21 MC38 tumours. **(F)** Comparison of PD-1 and TIM-3 subset compositions in p15E-reactive CD3^+^CD8^+^ TILs from D14 and D21 MC38 tumours. All data are representative of five biological replicates per timepoint. Graphs represent the mean (line) and standard error of the mean (SEM, error bars). Statistical analyses were performed using two-way ANOVA with *post-hoc* Dunnett's test, comparing tumour antigen peptide-reactive frequencies to irrelevant peptide controls **(B, C)**, or multiple unpaired Student's t-tests using the Holm-Sidak method to correct for multiple comparisons **(E, F)**. *p ≤ 0.05; **p ≤ 0.01; ***p ≤ 0.001.

The authors apologize for this error and state that this does not change the scientific conclusions of the article in any way. The original article has been updated.

