# Supplementary material for: Late-stage MC38 tumours recapitulate features of human colorectal cancer – implications for appropriate timepoint selection in preclinical studies
Source: Front Immunol. 2023 Apr 21;14:1152035. doi: 10.3389/fimmu.2023.1152035 (PMC10160415; doi:10.3389/fimmu.2023.1152035)
Supplement: Supplementary file 1 [file DataSheet_1.docx]

Supplementary Material

# Supplementary Methods

## Flow cytometry antibodies

**Supplementary Table 1.** Fluorophore-conjugated antibodies used in flow cytometry.

| Marker | Fluorophore | Antibody clone | Isotype | Manufacturer |
| --- | --- | --- | --- | --- |
| CD3 | FITC | 17A2 | Rat IgG2b, κ | BioLegend |
|  | BV421 | 17A2 | Rat IgG2b, κ | BioLegend |
| CD4 | PE/Cy7 | RM4-5 | Rat IgG2a, κ | BD Pharmingen |
|  | APC/H7 | GK1.5 | Rat IgG2b, κ | BD Pharmingen |
| CD8α | PE/Cy7 | 53-6.7 | Rat IgG2a, κ | BioLegend |
|  | AF700 | 53-6.7 | Rat IgG2a, κ | BioLegend |
|  | APC/H7 | 53-6.7 | Rat IgG2a, κ | BD Pharmingen |
| CD25 | BV421 | PC61 | Rat IgG1, λ | BioLegend |
| CD27 | PE | LG.3A10 | Armenian Hamster IgG | BD Pharmingen |
| CD28 | APC | 37.51 | Syrian Hamster IgG | BioLegend |
| CD39 | PE/Cy7 | Duha59 | Rat IgG2a, κ | BioLegend |
| CD44 | FITC | IM7 | Rat IgG2b, κ | BD Pharmingen |
| CD62L | PE/Cy7 | MEL-14 | Rat IgG2a, κ | BioLegend |
| CD69 | APC | H1.2F3 | Armenian Hamster IgG | BioLegend |
| CD127 (IL-7Rα) | PE/CF594 | SB/199 | Rat IgG2b, κ | BD Pharmingen |
| CD137 (4-1BB) | PE | 17B5 | Syrian Hamster IgG | BioLegend |
| CD223 (LAG-3) | PE | C9B7W | Rat IgG1, κ | BD Pharmingen |
| CD274 (PD-L1) | PE/Cy7 | 10F.9G2 | Rat IgG2b, κ | BioLegend |
| CD279 (PD-1) | FITC | 29F.1A12 | Rat IgG2a, κ | BioLegend |
|  | PerCP/Cy5.5 | 29F.1A12 | Rat IgG2a, κ | BioLegend |
| CD366 (TIM-3) | APC | B8.2C12 | Rat IgG1, κ | BioLegend |
|  | PE/Cy7 | B8.2C12 | Rat IgG1, κ | BioLegend |
| H-2K^b^ (MHC-I) | PerCP/Cy5.5 | AF6-88.5 | Mouse IgG2a, κ | BioLegend |
| EOMES | PE | DanIImag | Rat IgG2a, κ | eBioscience Inc. |
| FoxP3 | PE/Cy7 | FJK-16s | Rat IgG2a, κ | eBioscience Inc. |
| T-bet | APC | 4B10 | Mouse IgG1, κ | BioLegend |
| Granzyme B | PE/Cy7 | NG2B | Rat IgG2a, κ | eBioscience Inc. |
| IFNγ | APC | XMG1.2 | Rat IgG1, κ | BioLegend |
|  | AF700 | XMG1.2 | Rat IgG1, κ | BD Pharmingen |
| IL-2 | PE/CF594 | JES6-5H4 | Rat IgG2b, κ | BD Pharmingen |
|  | BV421 | JES6-5H4 | Rat IgG2b, κ | BD Pharmingen |
| IL-10 | PE | JES5-16E3 | Rat IgG2b, κ | BD Pharmingen |
|  | BV421 | JES5-16E3 | Rat IgG2b, κ | BioLegend |
| TNF | PE | MP6-XT22 | Rat IgG1, κ | BioLegend |

## IHC image processing and analysis

Quantitative evaluation of IHC images was performed according to published methodology (1) using ImageJ software (NIH). Images of complete tumour sections (TIFF format) were imported to ImageJ using the Bio-Formats plugin (2) and converted to RGB colour format. Subsequent image processing and analysis is described below.

***Definition of tumour regions and area quantitation***

To quantify the total surface area of tumour sections, the stromal–tumour interface in each image was defined visually using the Freehand Selection tool. Tumours were then divided into the outer and inner tumour regions (Supp. Fig. 1). The outer tumour region (from the tumour margin) was defined as the distal area 250 µm from the stromal–tumour interface, while the inner tumour region (tumour core) was defined as the inner area encompassed by the outer tumour region (Supp. Fig. 1). After setting the scale for each image, the area of each tumour region (as well as the total tumour area) was quantified in mm^2^ using the Analyse feature in ImageJ.

**
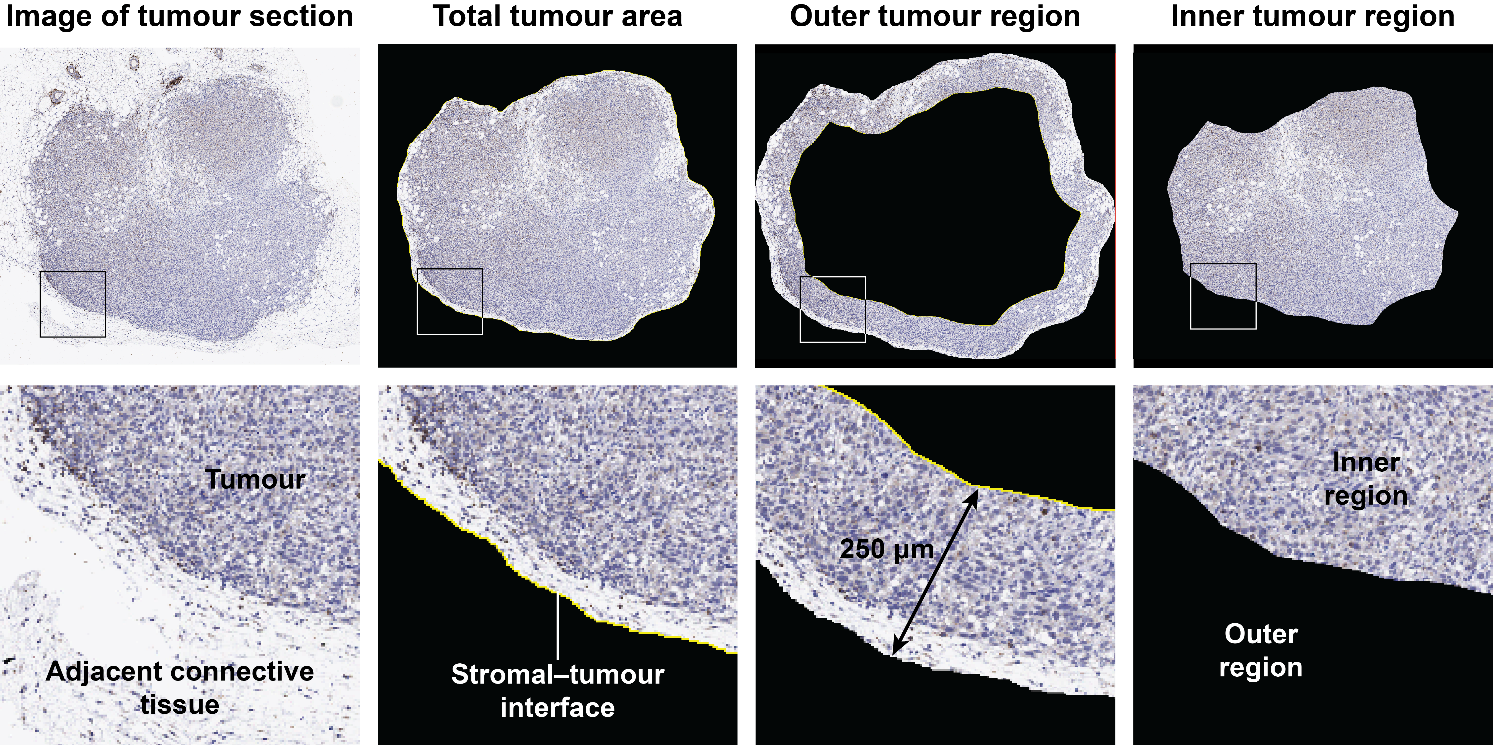
**

**Supplementary Figure 1. Definition of tumour regions and area quantitation**. For the quantification of tumour area, the stromal–tumour interface in each image was defined using the Freehand Selection tool in ImageJ (left images). Tumours were also divided into outer and inner tumour regions (right images). The outer tumour region was defined as the distal area 250 µm from the stromal–tumour interface, while the inner tumour region was defined as the inner area encompassed by the outer tumour region. The area of each tumour region (as well as the total tumour area) was quantified in mm^2^ using the Analyse feature in ImageJ. Masked images were then used for quantitative analyses. Representative images are derived from a tumour section stained for CD3.

***General image processing***

All IHC image processing consisted of four general phases: 1) background subtraction using the rolling ball algorithm method (with a 10 – 20 pixel radius; Supp. Fig. 2 A & 3 A); 2) spectral deconvolution to separate DAB and haematoxylin staining using the colour deconvolution plugin (3) (with the inbuilt haematoxylin and DAB vector; Supp. Fig. 2 B & 3 B); 3) thresholding to determine genuine DAB signals (Supp. Fig. 2 C & 3 C; using values optimised as described below); and 4) processing to remove staining artefacts and unwanted objects (object filling, Gaussian blur filtering and binarisation; Supp. Fig. 2 D – G & 3 D – G). Quantitative analysis of tumour vasculature (CD31 staining) and tumour infiltration by T cells (CD3, CD4 and CD8 staining) was subsequently performed after further (differential) image processing (described below).

***Optimisation of thresholding values***

The determination of intensity threshold represents a critical step in quantitative evaluation of IHC images (4). Hence, to validate automated image processing protocols, intensity values for thresholding were optimised for each IHC stain (CD3, CD4, CD8 and CD31). Briefly, 300 µm^2^ regions were randomly selected from three imaged sections (for each IHC stain) and the number of positive cells counted manually. Images were then processed with multiple 5-unit intensity iterations set for the thresholding of positive pixels (± the default thresholding value). Automated positive cell counts for each threshold iteration were then compared to the manual counts for each region. Thresholding values that most accurately enumerated positive cells after automated cell counting (within a range of <5% of manual counts) were applied to automated image processing protocols.

***Processing to remove staining artefacts and unwanted objects***

Following background subtraction, spectral deconvolution and thresholding, images were processed to remove staining artefacts; specifically “ring” staining artefacts caused by haematoxylin staining of cell nuclei (in CD3, CD4 and CD8 IHC images; Supp. Fig. 2 D & E, insets) and unstained vessel lumina (in CD31 IHC images; Supp. Fig. 3 D & E, insets). To enable positive cell counting and the quantitation of cross-sectional vessel area, respectively, these regions were masked using the “-Close” and “Fill holes” commands in ImageJ (Supp. Fig. 2 E & 3 E). Next, Gaussian blur filtering was applied to each image using a sigma radius value of 3 (Supp. Fig. 2 F & 3 F). To remove small staining artefacts, images were then converted to binary format (Supp. Fig. 2 G & 3 G). The resulting mask images were then subjected to quantitative analysis as detailed below.

***Quantitative analysis of T cell infiltration (CD3, CD4 and CD8 staining)***

To separate overlapping positive cells in CD3, CD4 and CD8 IHC analyses, mask images were subjected to the watershed segmentation algorithm in ImageJ (Supp. Fig. 2 G & H, insets). The absolute number of positive cells was then quantified using the “Analyse particles” feature (Supp. Fig. 2 I, inset). To exclude unwanted objects, the particle size range was set to 50 – 1000 units (pixels^2^) and the particle circularity range set to 0.2 – 1.0. After quantification, all image overlays were visually evaluated for consistency. Absolute cell numbers were then normalised to the corresponding tumour area (total tumour and the outer and inner regions) and expressed as the mean number of positive cells per mm^2^ (CD3^+^, CD4^+^ and CD8^+^ cells/mm^2^).


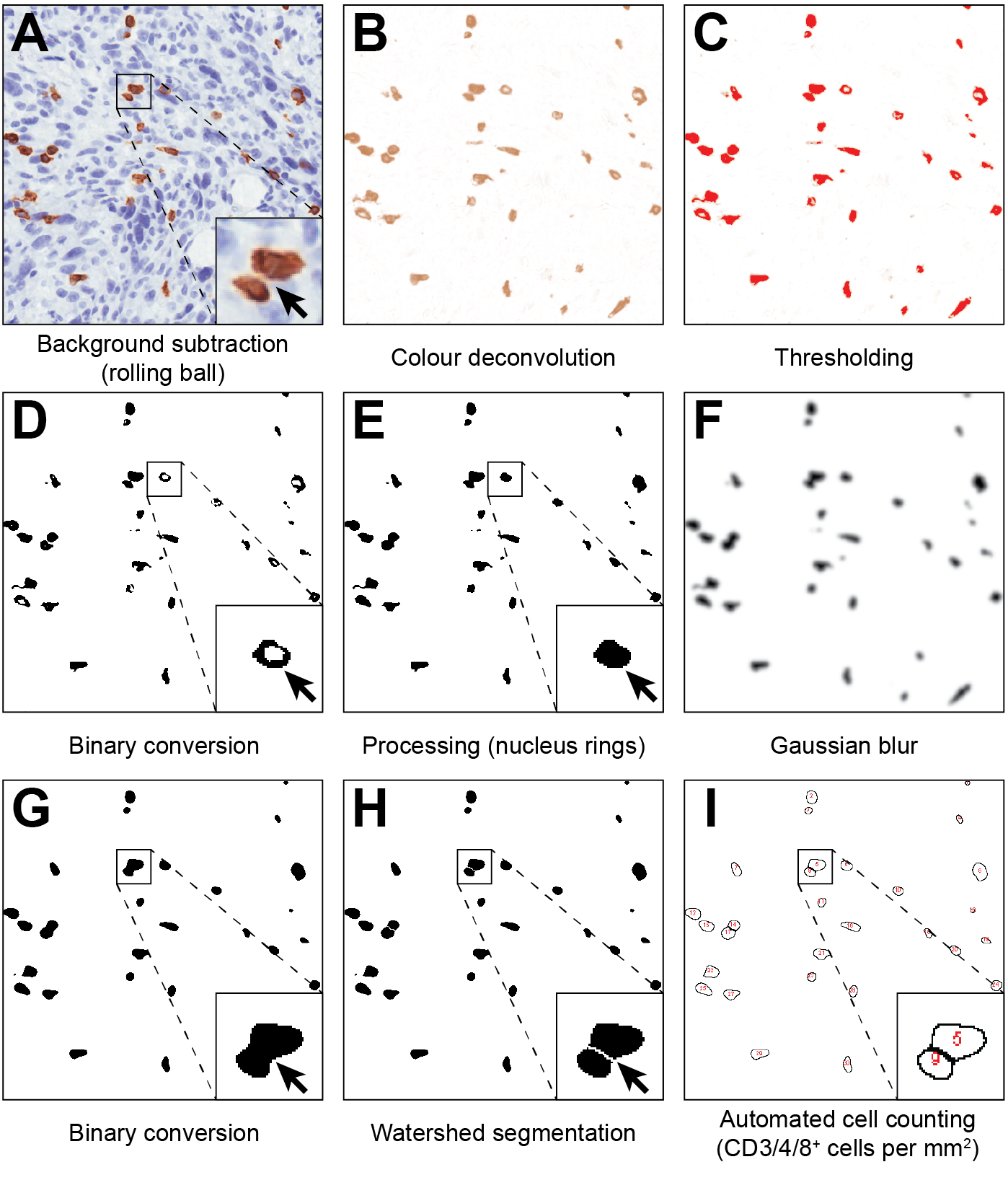


**Supplementary Figure 2. Quantitative analysis of T cell infiltration (****CD3, CD4 and CD8 immunohistochemistry).** For the quantification of CD3, CD4 and CD8 T cell infiltration, images were subjected to rolling ball background subtraction **(A)**, followed by colour deconvolution **(B)** and thresholding **(C)** to separate the DAB staining signal. To enable accurate enumeration of positive cells, “nucleus ring” staining artefacts (caused by haematoxylin staining of cell nuclei) were masked by further processing in ImageJ **(D & E, insets)**. To remove small staining artefacts, Gaussian blur filtering was applied to images **(F)**, before converting the images back to binary format **(G)**. To separate overlapping positive cells **(A, inset)**, mask images were then subjected to watershed segmentation **(G & H, insets)**. The absolute number of positive cells was then determined by automated counting **(I)** and normalised to the corresponding tumour area. Infiltrating T cell densities were expressed as the mean number of positive cells per mm^2^ (CD3^+^, CD4^+^ and CD8^+^ cells/mm^2^). Representative images are derived from a tumour section stained for CD4.

***Quantitative analysis of tumour vasculature (CD31 staining)***

Quantitative analysis of tumour vasculature (CD31 staining) was performed on mask images only (no watershed segmentation). The “Analyse particles” feature was used to quantify the absolute number of CD31^+^ objects, the total CD31^+^ area (µm^2^), and the mean size of CD31^+^ objects (µm^2^). For the quantification of the total CD31^+^ area (µm^2^), large CD31^+^ vessels and individual CD31^+^ cells were included, with the particle size range set to 50 – ∞ units (pixels^2^) and no circularity criteria (as shown in Supp. Fig. 3 H). For the quantification of vessel density (CD31^+^ objects), individual CD31^+^ cells were excluded by increasing the minimum particle size threshold (as shown in Supp. Fig. 3 I). After quantification, all image overlays were visually evaluated for consistency. CD31^+^ object counts and total CD31^+^ area measurements were then normalised to the corresponding tumour area (total tumour and the outer and inner regions). Vessel density was expressed as the absolute number of CD31^+^ blood vessels per mm^2^ (CD31^+^ objects/mm^2^) and vascularity was expressed as the CD31^+^ area of the corresponding tumour area (%CD31^+^ area). Mean vessel size was quantified as the mean area covered by CD31^+^ objects (including the vessel lumen; µm^2^) within designated tumour regions.


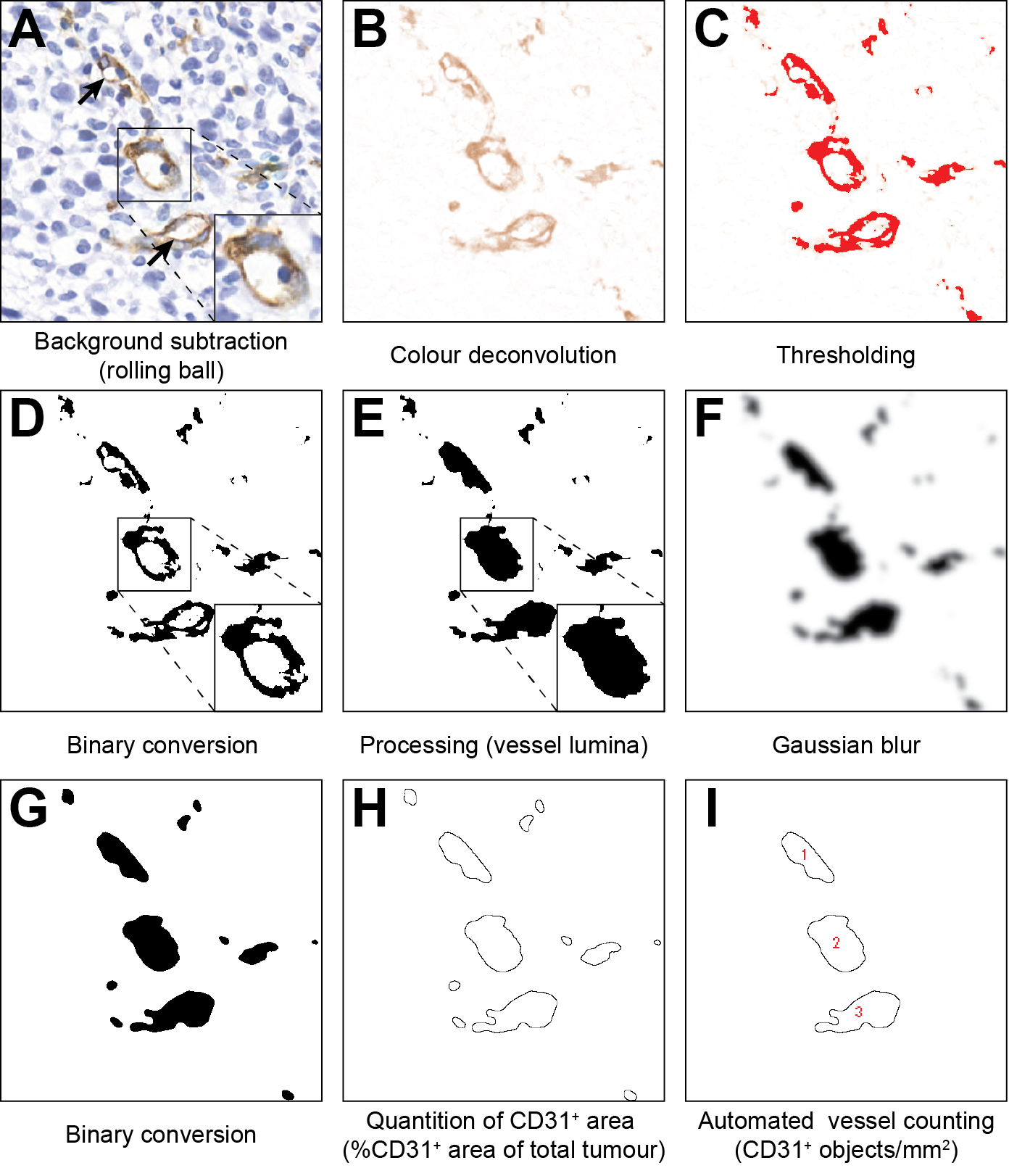


**Supplementary Figure 3. Quantitative analysis of tumour vasculature (CD31 immunohistochemistry).** For the analysis of CD31 IHC, images were subjected to rolling ball background subtraction **(A)**, followed by colour deconvolution **(B)** and thresholding **(C)** to separate the DAB staining signal. To enable the quantitation of cross-sectional vessel area, unstained vessel lumina (indicted by arrows in **A**) were masked by further processing in ImageJ **(D & E, insets)**. To remove small staining artefacts, Gaussian blur filtering was applied to images **(F)**, before converting the images back to binary format **(G)**. The resulting mask images were then subjected to quantitative analysis. For the quantification of CD31^+^ area (%CD31 area of total tumour), large CD31^+^ vessels and individual CD31^+^ cells were included **(H)**. For the quantification of vessel density (CD31^+^ objects/mm^2^), individual CD31^+^ cells were excluded and the number of large CD31^+^ objects (vessels) determined by automated counting.

## Detection of neoantigen mutations

Regions encompassing nonsynonymous mutations in *Adpgk*, *Dpagt1* and *Reps1* of the MC38 genome (reported by Yadav *et. al.* (5)) were selectively amplified by polymerase chain reaction (PCR). Primer sets flanking these mutations were designed using the open-source Primer3 software (6, 7) and submitted to Primer-BLAST (NCBI, Bethesda, MD, USA) to exclude non-specific primer sets. Selected primer pairs are detailed below (Supplementary Table 1) and were obtained from Integrated DNA Technologies (IDT Coralville, IA, USA).

**Supplementary Table 2.** PCR primers used to amplify regions encompassing MC38 neoantigen mutations

| Target gene | Forward primer (5’-3’) | Reverse primer (5’-3’) | Product size |
| --- | --- | --- | --- |
| *Adpgk* | TTCCTGAGGATGCAAAGGGT | TTAGTGGACCTGTCAGAGCC | 283 bp |
| *Dpagt1* | GTCCCAGCCTTCCAAGTAGT | TCATTCCCCACCCCTTACAC | 388 bp |
| *Reps1* | GAGCGACGCCGAGCAGAAAT | TGCACACACAACCCGAGAGACC | 359 bp |

Genomic DNA (gDNA) was extracted from MC38 tumour cells and wildtype C57BL/6 splenocytes using the QIAamp DNA Blood Mini kit (Qiagen, Hilden, Germany) according to the manufacturer’s instructions. PCR was performed using Platinum *Taq* DNA polymerase (Invitrogen, Carlsbad, CA, USA) and components provided by the manufacturer. Reactions were performed in a total volume of 25 μL and contained 1x PCR buffer; 0.8 mM deoxyribonucleotide triphosphate (dNTP) mixture (0.2 mM per dNTP); 1.5 mM MgCl_2_; 0.2 μM forward and reverse primer; template DNA (1 – 4 ng) and 2U Platinum *Taq* DNA polymerase (in MilliQ H_2_O). Thermal cycling for PCRs was performed in a T100 thermal cycler (Bio-Rad Laboratories, Hercules, CA, USA) as follows: 94°C for 2 minutes (pre-denaturation); 35 cycles of 94°C for 30 seconds (denaturation), 57°C for 30 seconds (annealing), 72°C for 1 minute (extension); and 72°C for 5 minutes (final extension). For *Reps1* amplification where standard conditions proved unsuitable, 9% KB extender (Invitrogen) was added to the reaction mix and the annealing temperature during thermal cycling was increased to 63°C.

PCR products were subjected to agarose gel electrophoresis (AGE) to determine successful amplification. 2% w/v agarose gels were prepared by dissolving agarose powder (Bio-Rad Laboratories) in 0.5× Tris Acetate-EDTA (TAE) buffer (20 mM Tris base, 2.5 mM CH_3_COONa, 0.5 mM Na_2_ EDTA, adjusted to pH 7.9 with glacial acetic acid at 10×; all from Sigma-Aldrich). 600 nM ethidium bromide (Sigma-Aldrich) was incorporated into gels to allow DNA visualisation. PCR products were combined with 2 µL BlueJuice gel loading buffer (Invitrogen) and 5 µL of each sample loaded per well. Gels were electrophoresed for 25 minutes at 135 V and then imaged using a Molecular Imager Gel Doc XR+ transilluminator system and Image Lab software (both from Bio-Rad Laboratories). DNA band sizes were assessed using 1 Kb Plus DNA ladder (Invitrogen).

PCR amplification products were purified and concentrated for sequencing using the DNA Clean and Concentrator-5 kit (Zymo Research, Irvine, CA, USA) according to the manufacturer’s instructions. Purified PCR amplicons (supplied as 4 ng template DNA with 0.4 µM forward or reverse primer in MilliQ H_2_O) were sequenced using the fluorescence-based Sanger method by University of Otago Genetic Analysis Services (Department of Anatomy, University of Otago, Dunedin, NZ). Chromatograms of DNA sequences were analysed using Chromas Lite software (Technelysium, Brisbane, QLD, Australia). Single-nucleotide variant (SNV) mutations were identified by comparing the amplicon sequences obtained from MC38 tumour cells with the corresponding wildtype amplicon sequences obtained from C57BL/6 splenocytes.

# Supplementary Data

## Supplementary Figures


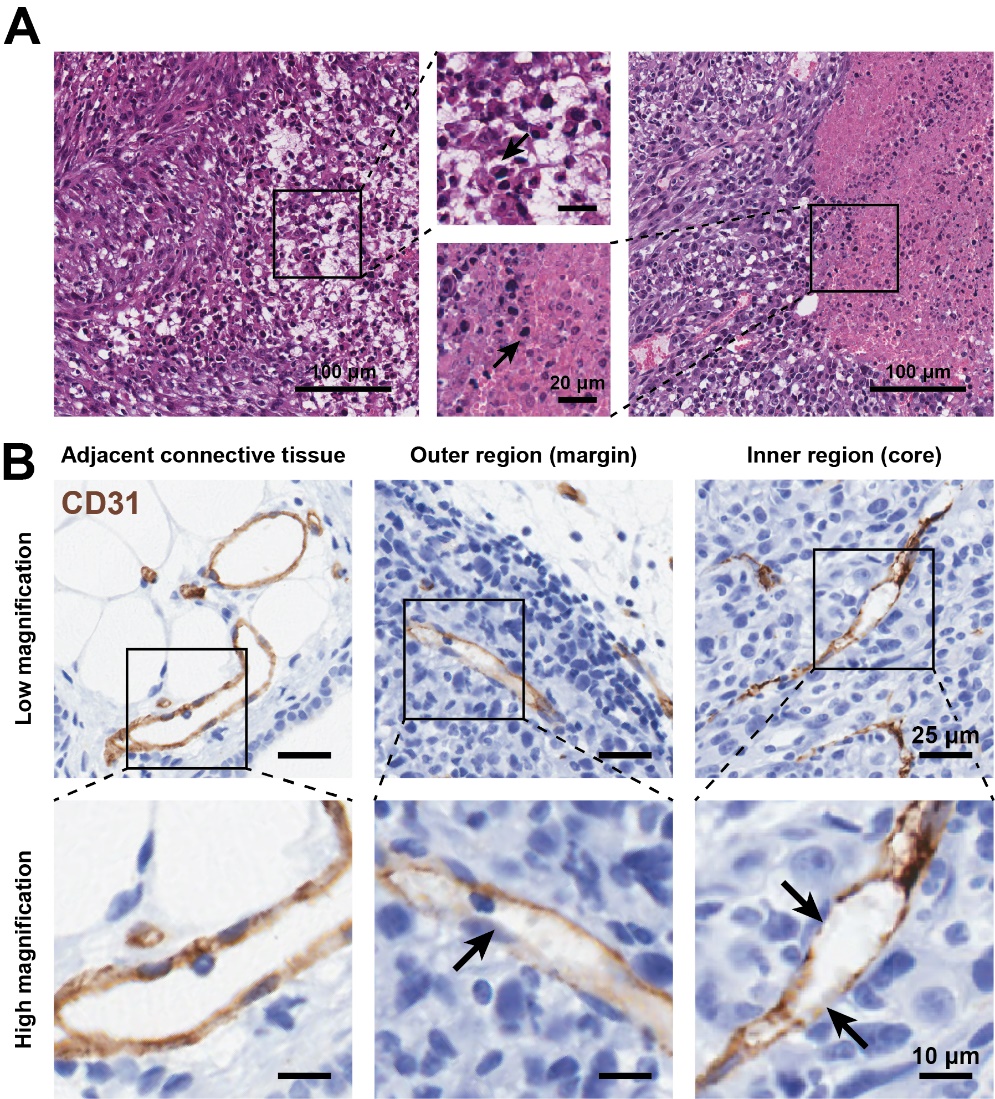


**Supplementary Figure 4. Necrosis and abnormal vasculature in MC38 tumours. (A)** H&E images depict focal areas of ischemic (left) and haemorrhagic (red blood cell extravasation; right) necrosis in Day 14 MC38 tumours. Insets depict tumour nuclei undergoing pyknosis (chromatin condensation; arrows), karyolysis (chromatin degradation) and karyorrhexis (nuclear fragmentation). **(B)** Vascular abnormalities were assessed in formalin-fixed, paraffin-embedded tumour sections by CD31 immunohistochemistry (brown) with haematoxylin counterstain (blue). Images depict CD31^+^ vessel structure in adjacent connective tissue and the outer (margin) and inner (core) regions of Day 21 MC38 tumours. Note the relatively normal vessel structure in adjacent connective tissue, characterised by intact CD31^+^ endothelium supported by fine, perivascular connective tissue. By contrast, CD31^+^ vessels in both the outer and inner regions of MC38 tumours exhibit aberrant endothelium which is either attenuated in thickness or entirely absent (arrows in enlarged insets). All scale bars depict distances denoted in the right-sided images (μm). All data are representative of 3-4 biological replicates per timepoint.


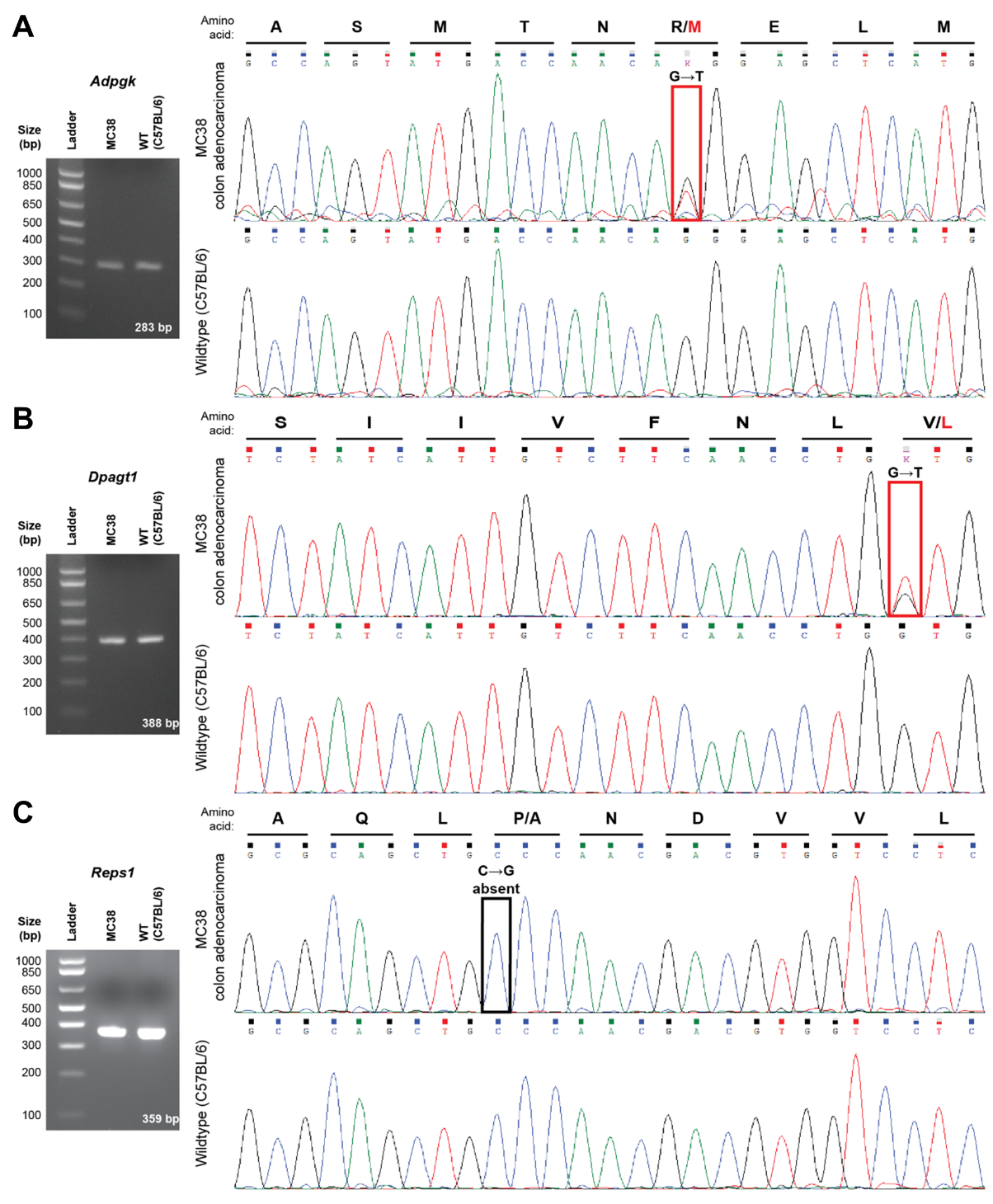


**Supplementary Figure 5. Necrosis and abnormal vasculature in MC38 tumours.** Genomic DNA (gDNA) was isolated from MC38 tumour cells and wildtype C57/BL6 splenocytes (WT). PCR was then performed using primer pairs encompassing each of the mutated regions reported by Yadav *et. al.* (5). To confirm the presence of neoantigen mutations in MC38 tumour cells, the resulting PCR products were subjected to Sanger sequencing. PCR products visualised on agarose gels following electrophoresis (left) and sequencing results (right) for *Adpgk* **(A)***, Dpagt1* **(B)** and *Reps1* **(C)**. In MC38, the reported non-synonymous point mutations are present in *Adpgt* and *Dpagt1* (red boxes, both G→T, heterogeneous), but absent in *Reps1* (black box). The MHC class I-restricted neo-peptide sequences are indicated above, showing the arginine to methionine (R/M) and valine to leucine (V/L) amino acid alterations in *Adpgk* and *Dpagt1*, respectively. Abbreviations: PCR, polymerase chain reaction; bp, base pairs.

1. **References**

1. Väyrynen JP, Vornanen JO, Sajanti S, Böhm JP, Tuomisto A, Mäkinen MJ. An improved image analysis method for cell counting lends credibility to the prognostic significance of T cells in colorectal cancer. Virchows Archiv. 2012;460(5):455-65.

2. Linkert M, Rueden CT, Allan C, Burel J-M, Moore W, Patterson A, et al. Metadata matters: access to image data in the real world. Journal of Cell Biology. 2010;189(5):777-82.

3. Ruifrok AC, Johnston DA. Quantification of histochemical staining by color deconvolution. Analytical and quantitative cytology and histology. 2001;23(4):291-9.

4. Varghese F, Bukhari AB, Malhotra R, De A. IHC Profiler: an open source plugin for the quantitative evaluation and automated scoring of immunohistochemistry images of human tissue samples. PloS one. 2014;9(5):e96801.

5. Yadav M, Jhunjhunwala S, Phung QT, Lupardus P, Tanguay J, Bumbaca S, et al. Predicting immunogenic tumour mutations by combining mass spectrometry and exome sequencing. Nature. 2014;515(7528):572-6.

6. Untergasser A, Cutcutache I, Koressaar T, Ye J, Faircloth BC, Remm M, et al. Primer3—new capabilities and interfaces. Nucleic acids research. 2012;40(15):e115-e.

7. Koressaar T, Remm M. Enhancements and modifications of primer design program Primer3. Bioinformatics. 2007;23(10):1289-91.
